# Hypocrystalline ceramic aerogels for thermal insulation at extreme conditions

**DOI:** 10.1038/s41586-022-04784-0

**Published:** 2022-06-29

**Authors:** Jingran Guo, Shubin Fu, Yuanpeng Deng, Xiang Xu, Shujin Laima, Dizhou Liu, Pengyu Zhang, Jian Zhou, Han Zhao, Hongxuan Yu, Shixuan Dang, Jianing Zhang, Yingde Zhao, Hui Li, Xiangfeng Duan

**Affiliations:** 1 Key Lab of Smart Prevention and Mitigation of Civil Engineering Disasters of the Ministry of Industry and Information Technology and Key Lab of Structures Dynamic Behavior and Control of the Ministry of Education, Harbin Institute of Technology, Harbin, China; 2 Department of Chemistry and Biochemistry, University of California, Los Angeles, CA USA

**Keywords:** Metamaterials, Ceramics, Gels and hydrogels

## Abstract

Thermal insulation under extreme conditions requires materials that can withstand complex thermomechanical stress and retain excellent thermal insulation properties at temperatures exceeding 1,000 degrees Celsius^1–3^. Ceramic aerogels are attractive thermal insulating materials; however, at very high temperatures, they often show considerably increased thermal conductivity and limited thermomechanical stability that can lead to catastrophic failure^4–6^. Here we report a multiscale design of hypocrystalline zircon nanofibrous aerogels with a zig-zag architecture that leads to exceptional thermomechanical stability and ultralow thermal conductivity at high temperatures. The aerogels show a near-zero Poisson’s ratio (3.3 × 10^−4^) and a near-zero thermal expansion coefficient (1.2 × 10^−7^ per degree Celsius), which ensures excellent structural flexibility and thermomechanical properties. They show high thermal stability with ultralow strength degradation (less than 1 per cent) after sharp thermal shocks, and a high working temperature (up to 1,300 degrees Celsius). By deliberately entrapping residue carbon species in the constituent hypocrystalline zircon fibres, we substantially reduce the thermal radiation heat transfer and achieve one of the lowest high-temperature thermal conductivities among ceramic aerogels so far—104 milliwatts per metre per kelvin at 1,000 degrees Celsius. The combined thermomechanical and thermal insulating properties offer an attractive material system for robust thermal insulation under extreme conditions.

## Abstract (web summary)

Hypocrystalline ceramic aerogels with a zig-zag architecture show high thermal stability under thermal shock and exposure to high temperature, providing a reliable material system for thermal insulation at extreme conditions.

## Main

Thermal insulation under extreme conditions, such as complex mechanical loadings and large thermal gradients in deep-space and deep-earth environments, requires reliable structural stability and exceptional insulating capability. Ceramic aerogels exhibit a super-insulating capability with thermal conductivity (*κ*) less than that of stationary air (26 mW m^−1^ K^−1^)^1^. However, owing to their intrinsic brittle nature, ceramic aerogels usually suffer from serious strength degradation and structural collapse under large mechanical stresses or thermal shocks^2–4^, which may severely compromise thermal insulation properties and lead to catastrophic failure under extreme conditions. Therefore, retaining robust structural stability under extreme conditions represents a critical challenge for reliable thermal insulation.

Studies so far have shown that the mechanical properties of ceramic aerogels can be considerably enhanced through elaborate structural engineering^1,5–20^. For example, ceramic aerogels constructed from one-dimensional fibres can form a flexible porous framework by physical twining or chemical bonding, which can result in not only compression elasticity but also, more importantly, additional stretchability and bendability^21,22^. However, owing to the randomly twined structure and dislocation between adjacent fibres, fibrous ceramic aerogels^23–26^ have exhibited elastic compressibility only up to 80% strain, yet still with considerable structural degradation. To address this challenge, specifically designed structures with a tailored Poisson’s ratio (*ν*)^27^ may substantially enhance the performance metrics^19^. In particular, a near-zero *ν* could help minimize or eliminate the excessive stress induced by the longitudinal deformation, and achieve a near-zero transverse strain, which may offer optimal mechanical flexibility in fibrous structures.

Besides the mechanical flexibility, thermal stability is another crucial factor in designing ceramic aerogels for thermal insulation under extreme conditions. Owing to the crystallization-induced pulverization, insufficient oxidation resistance and large thermal expansion coefficient (*α*) behaviour under large thermal gradients or high-temperature exposure, ceramic aerogels often exhibit weak thermal stability with serious strength degradation and structural collapse^6^. The crystallization inhibition and oxidation resistance can be improved by tuning the elemental composition or crystalline structure of the ceramic materials^11,24,26,28^. However, the thermal expansion behaviour is more difficult to regulate when coupled with the mechanical properties and structural geometric configurations. Inspired by the negative-*α* strategy^29,30^, ceramic aerogels with a near-zero *α* can further eliminate the mismatched component strain and suppress the thermomechanical stress to ensure superior thermal stability.

Reliable thermal insulation under extreme conditions requires an unusual combination of mechanical flexibility, high thermal stability and low thermal conductivity across a wide temperature range. Some of these critical material parameters exhibit intrinsic trade-offs with each other for most ceramic aerogels^13,31^. For example, the ultralow-density feature of typical ceramic aerogels is beneficial for greatly suppressed solid conduction to achieve ultralow *κ* (26 mW m^−1^ K^−1^ to 33 mW m^−1^ K^−1^ in air^11,13,23,26^) at room temperature, but substantially increases thermal radiation heat transfer and leads to much higher *κ* at high temperature (for example, over 1,000 °C), which is clearly more relevant for extreme-condition operations. In particular, thermal radiation scales linearly with the reciprocal of material density and the third power of kelvin temperature, which becomes dominant thermal conduction at temperatures over 500 °C. Most ceramic aerogels exhibit a rather high *κ* of about 200 mW m^−1^ K^−1^ around 1,000 °C owing to their low density and low infrared radiation absorption^6^. A potential strategy to reduce thermal radiation heat transfer is to incorporate infrared radiation absorbing and reflecting materials such as carbon or titanium dioxide. However, carbon can usually be thermally etched at high temperatures, whereas titanium dioxide can reduce aerogel structural stability owing to its softening effect^32^. Therefore, it is a challenge to realize excellent high-temperature thermal insulating performance while retaining robust thermomechanical stability in typical ceramic aerogels.

Here we report a multiscale design and synthesis of hypocrystalline zircon nanofibrous aerogels (ZAGs) with a zig-zag architecture, to realize near-zero *ν* and near-zero *α* for superior thermomechanical properties. The resulting ZAGs feature high mechanical flexibility, high thermal stability under sharp thermal shocks and high-temperature exposures, and exceptional high-temperature thermal insulating performance (104 mW m^−1^ K^−1^ at 1,000 °C and 26 mW m^−1^ K^−1^ at 25 °C and in air), presenting a reliable material for thermal insulation under extreme conditions.

## Design rationale

**Fig. 1:**
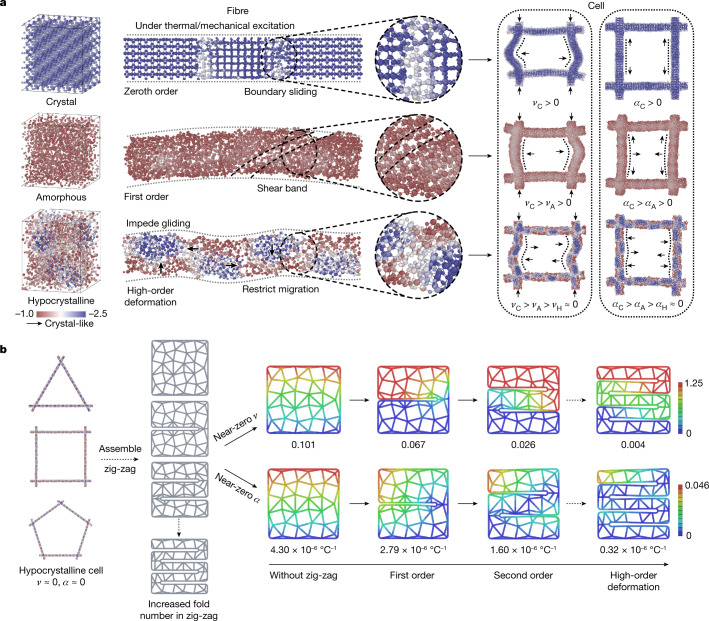
Multiscale design of hypocrystalline ceramic nanofibrous aerogel. **a**, Deformation modes and the corresponding *ν* and *α* of crystalline (C), amorphous (A) and hypocrystalline (H) ceramic fibrous cells under mechanical and thermal excitations. The coloured scale bar indicates the variation of ceramics from amorphous to crystal by using a local-entropy-based fingerprint to characterize the crystallinity of each atom in the simulated system. **b**, Illustration of the zig-zag architecture design based on hypocrystalline fibrous ceramics. The units of the coloured scale bars are millimetres, presenting absolute displacement values in *ν* and *α* calculation. The triangle, square and pentagon cells are the building units to assemble the fibrous aerogel structure.

Thermal insulation under extreme conditions requires ceramics that can withstand complex mechanical loadings as well as sharp thermal shocks. Typical crystalline ceramics exhibit intrinsic brittleness owing to their weak dislocation and grain boundary sliding and softening effects^33^. Amorphous ceramics may show improved deformability by triggering shear bands^34^, but often suffer from the localization of shear strain and easy crystallization at high temperatures. To address these challenges, we design a hypocrystalline (nanocrystals embedded in an amorphous matrix) ceramic, with an amorphous matrix as grain boundaries to impede the gliding of nanocrystalline domains, and nanocrystalline domains as the staples to restrict the migration of the amorphous matrix at high temperatures (Fig. 1a, Supplementary Fig. 1), to achieve superior thermomechanical properties. With this hypocrystalline design, the deformation of a ceramic fibre under mechanical and thermal excitations features a high-order buckling mode instead of a uniform pattern, thus providing additional degrees of freedom to facilitate the high-order deformation mode, reducing the *ν* and *α* of the fibrous cell to near-zero values (Fig. 1a). In addition, a well designed architecture can extend the near-zero *ν* and near-zero *α* from local cells to the global structure^35^. To this end, the hypocrystalline fibres were further assembled into a zig-zag architecture for additional high-order deformation modes at the macroscale level to endow near-zero *ν* and near-zero *α* in macroscopic ceramic aerogels (Fig. 1b, Supplementary Fig. 2).

## Ceramic aerogel fabrication

**Fig. 2:**
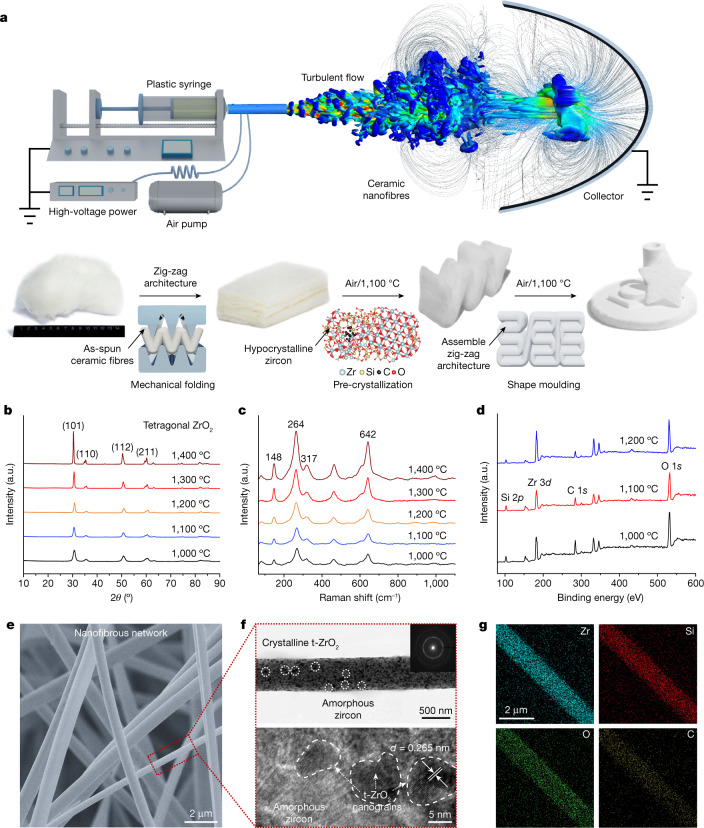
Fabrication process and material characterization of hypocrystalline ZAGs. **a**, Illustration of the turbulent-flow-assisted electrospinning and fabrication process of ZAGs. **b**–**d**, XRD (**b**), Raman (**c**) and XPS (**d**) of ZAGs. **e**, SEM image of the nanofibrous network in aerogel. **f**, TEM and high-resolution TEM images of a zircon nanofibre with hypocrystalline zircon and the corresponding selected-area electron diffraction (inset). **g**, EDS mapping images of a single nanofibre.

We first fabricated a three-dimensional (3D) zircon fibrous matrix by an electrospinning method in an air turbulent flow field (Fig. 2a). The conventional electrospinning method tends to form a two-dimensional (2D) non-woven membrane with high density owing to the electrostatic ejection and fibre aggregation on the collector^36,37^. To this end, we introduced a coaxial air-blowing setup into the regular electrospinning device to electrospin the zirconium–silicon precursor. High-speed air blown out from the outer nozzle first formed a jet and then transited into the turbulence behind the Taylor cone, resulting in a complex 3D turbulent flow field (Supplementary Figs. 3, 4). This turbulent field could overcome the electrical orientation effect, and move the resulting nanofibres in a complex trajectory to entangle with each other and form a randomly twined fibrous aerogel structure (Supplementary Figs. 5, 6). Next, we used a mechanical folding process (Supplementary Fig. 7) to produce a zig-zag architecture in the as-spun fibrous aerogels and mitigate the architecture-induced increase of *ν* and *α* (Supplementary Fig. 2). Lastly, we prepared the hypocrystalline ZAGs through a simple pre-crystallization process by thermal annealing at 1,100 °C in air. A secondary sintering treatment at 1,100 °C in air was used to crosslink zig-zag units for a large-scale, low-cost and diverse-shape specimen (thick layer, pentagram, circular tube and so on) (Fig. 2a, Supplementary Figs. 5–8, 22, 23). The resulting ZAGs exhibit low densities of about 15–55 mg cm^−3^ (Supplementary Fig. 7), establishing them as a member of superlight solid materials. All tests were done on aerogels with a density of 20 mg cm^−3^ unless otherwise noted.

## Characterization

We characterized the chemical composition and crystallinity of ZAGs using X-ray diffraction (XRD), Raman spectra and X-ray photoelectron spectroscopy (XPS) (Fig. 2b–d). XRD studies revealed a high-crystallinity tetragonal zirconium dioxide structure. In particular, the characteristic XRD and Raman peaks show increasingly narrower width and higher intensity with higher annealing temperature, indicating gradually increasing crystallization. Scanning electron microscopy (SEM) studies showed the zig-zag architecture with representative corner joints and parallel layers developed from the randomly twined fibrous network (Fig. 2e, Supplementary Fig. 9; fibre diameter around 700 nm, Supplementary Fig. 10). After pre-crystallization treatment at 1,100 °C, the entangled ZAG nanofibres were further bonded with each other by additional chemical crosslinking (Supplementary Fig. 9), which enhances the overall structural stability. The transmission electron microscopy (TEM) and selected-area electron diffraction (Fig. 2f, Supplementary Figs. 11, 12) further showed that the ceramic nanofibres consist of polycrystalline zirconium dioxide nanograins (smaller than 10 nm) embedded in an amorphous zircon matrix, indicating a hypocrystalline structure. The transformation of amorphous zircon to hypocrystalline zircon upon high-temperature annealing was also confirmed from the well tempered metadynamics (WTMetaD)^38^ simulation (Supplementary Figs. 13, 14). Together, our studies demonstrate the formation of unique hypocrystalline ZAGs, in which the amorphous zircon functions as a binder to crosslink tetragonal zirconium dioxide in and between the nanofibres.

Interestingly, the XPS (Fig. 2b) and energy-dispersive X-ray spectroscopy mapping (EDS) results (Fig. 2g) indicate that some residual amorphous carbon was well retained in the hypocrystalline zircon even after the thermal treatment at high temperatures (1,100 °C), which is attributed to some residue carbon trapped in zircon nanofibres during the pre-crystallization of the as-spun fibrous aerogels (Supplementary Fig. 15). The apparent off-white (light greyish) colour of the resulting ZAGs also indicates the presence of carbon (Supplementary Fig. 15). The retainment of the carbon in the zircon nanofibres after such a high-temperature process is intriguing and has important implications for reducing thermal radiation heat transfer (see thermal conductivity studies below). Molecular dynamics (MD) simulations (Supplementary Figs. 16, 17) suggested that the tetragonal zirconium dioxide nanograins have a critical role in restricting the flow of the amorphous zircon at high temperatures, and revealed that the entrapped amorphous carbon was well protected from oxidative etching at 1,100 °C when the thickness of the pinned amorphous zircon was greater than 1.5 nm.

## Mechanical properties

**Fig. 3:**
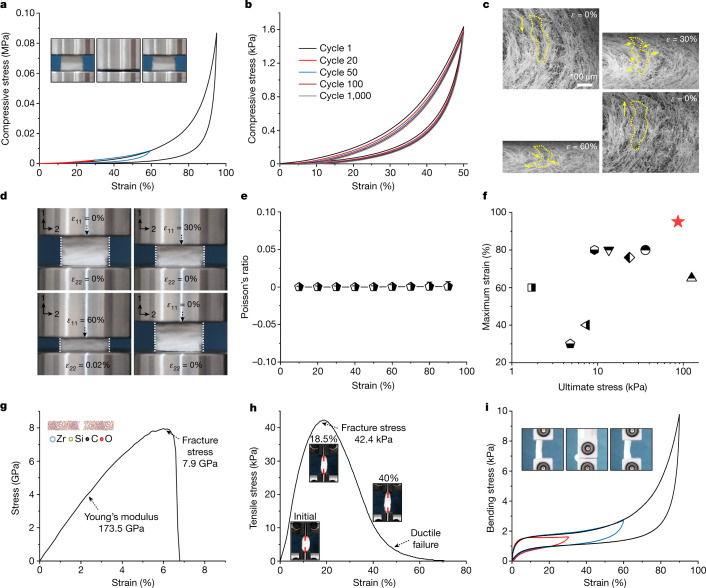
Mechanical properties of hypocrystalline ZAGs. **a**, Uniaxial compression of ZAGs with strain up to 95%. The red, blue and black curves represent compressive strain of 30%, 60% and 95%, respectively. Inset: experimental snapshots of one compression cycle. **b**, The compressive stress–strain curves of ZAGs at 50% strain for 1,000 cycles. **c**, In situ SEM images of ZAGs under uniaxial compression. *ε* is the in situ compressive strain. **d**, Experimental snapshots of the near-zero *ν* behaviour of ZAGs under uniaxial compression. **e**, The near-zero *ν* of ZAGs under different compressive strains. Standard deviations of *ν* are shown as error bars. **f**, The maximum compressive strain and ultimate stress of ZAGs compared with other ceramic nanofibrous aerogels. Red pentagram, ZAGs in this work; square, Si_3_N_4_ nanobelt^11^; pentagon, oxide ceramic fibre^12^; bottom triangle, Al_2_O_3_ nanotube^31^; hexagon, SiO_2_ fibre with SiO_2_ nanoparticles^10^; upside-down triangle, SiO_2_–Al_2_O_3_ fibre^26^; diamond, SiC nanowire^40^; circle, PAN/BA-a and SiO_2_ fibre^14^; right-side-up triangle, BN nanotube^18^. **g**, The stress–strain curve from the MetaD simulation of the single nanofibre under uniaxial tension. Inset: fracture simulation of single fibre. **h**, The tensile stress–strain curve of ZAGs. Inset: experimental snapshots of tensile fracture. **i**, The bending test of ZAGs with strain up to 90%. The red, blue and black curves represent bending strain of 30%, 60% and 90%, respectively. Inset: experimental snapshots of the bending experiment.

We next investigated the mechanical properties of ZAGs with quasi-static compression, tension, bend and torsion. For the compression test, the sample was compressed from 10 mm to 0.5 mm, a strain of 95% (to our knowledge, the largest value so far), and completely recovered the original configuration after pressure release (Fig. 3a, Supplementary Fig. 18, Supplementary Video 1). The recoverable strain is notably higher than previously reported values for ceramic nanofibrous aerogels, which tops out at 80% (refs. ^13,14,31,39–43^). The ZAGs could be repeatedly compressed at 50% strain for 1,000 cycles with little stress degradation, less than 7%. The 1,000th cycle loop remained nearly unchanged compared with the 100th cycle, and the height of the sample remained nearly unchanged (residual strain <5%) (Fig. 3b, Supplementary Video 2), indicating an excellent fatigue resistance (Supplementary Figs. 19–21). This superior compressive elasticity can be attributed to the multiscale enhancement in our ZAGs. The hypocrystalline zircon components (Fig. 2f) ensure a fundamental toughening in the material attributed to the amorphous zircon binders with near-ideal strength from the strain-gradient theory^44,45^, whereas the nanofibre building blocks provide an excellent deformability (Supplementary Fig. 24) and the connected fibrous network (Supplementary Fig. 9) improves the elasticity by reducing the plastic deformation induced by the dislocation between adjacent fibres. In addition, the zig-zag architecture (Fig. 1, Supplementary Figs. 1, 2) endows a superelasticity (Fig. 3c) with near-zero *ν* (3.3 × 10^−4^) behaviour (Fig. 3d, e). This near-zero *ν* can effectively reduce plastic deformation by restricting the local and global indirect tension failures and enhancing the deformation compatibility. Moreover, the bending moments of curved corner joints in the zig-zag architecture can provide additional resilience to facilitate the aerogel to recover from compression with less residual strain. For ZAGs, the ultimate compressive stress is up to 86.8 kPa compared with 13.5 kPa for silicon dioxide (SiO_2_)–aluminium oxide (Al_2_O_3_) fibrous aerogels, and the maximum strain is 95% compared with 80% for SiO_2_ fibrous aerogels (Fig. 3f)^13,26^.

We next used MD simulations to evaluate the mechanical properties of a single hypocrystalline zircon nanofibre. The simulated tensile stress–strain curve reveals that the Young’s modulus, tensile strength and strain of a single hypocrystalline zircon nanofibre are 173.5 GPa, 7.9 GPa and 6.7%, respectively (Fig. 3g). This unusual tensile enhancement can be attributed to the near-ideal strength of the amorphous matrix with impeded gliding of the crystals (Supplementary Figs. 17, 25), which improves the stretchability of ZAGs. We next experimentally evaluated the stretchability of ZAGs with uniaxial quasi-static tension, revealing that the ZAGs can withstand a nearly linear tensile strain up to 18.5% followed by a nonlinear tensile strain up to 40% (Fig. 3h, Supplementary Video 3), which is comparable to the highest values reported so far^13,21,22,41,42^ (Supplementary Fig. 26). The tensile fracture stress reached up to 42.4 kPa. The high tensile ductility can be attributed to a synergistic effect from the nanoscopic hypocrystalline zircon and the microscopically well crosslinked nanofibrous network^22,46,47^. The bendability test using a typical two-point buckling method revealed that the ZAGs show excellent flexibility with a bending strain up to 90% without any external fracture failure (Fig. 3i, Supplementary Fig. 27, Supplementary Video 4). Lastly, the torsional property studies with a unilateral torsion device showed that the ZAGs exhibited a torsional angle up to 360°, and an exceptional fatigue resistance with little morphology change or structural degradation under cyclic loadings (Supplementary Fig. 28).

## Thermal stability

**Fig. 4:**
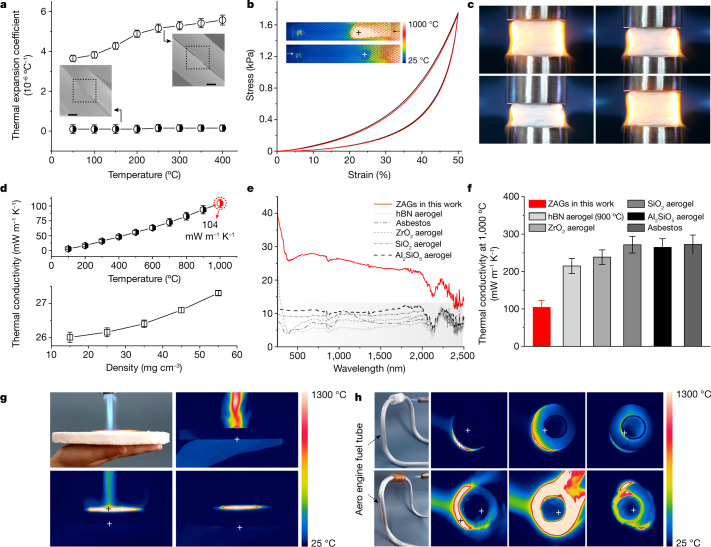
Thermal stability and thermal insulation properties of ZAGs. **a**, The near-zero *α* (black circle) of ZAGs compared with the positive *α* (white circle) under different temperatures. Inset: the corresponding SEM images of crosslinking state among nanofibres. Error bars indicate standard deviations of *α*. Scale bars = 200 nm. **b**, The strain and stress evolution after 10,000 cycles of sharp thermal shocks (200 °C s^−1^). The black and red curves represent the mechanical performance (50% compressive strain) after the 1st and 10,000th cycles of thermal shocks. Inset: infrared images of ZAGs in one thermal shock cycle. **c**, Compression and recovery process under 50% compressive strain of the ZAGs in the bilateral flame of a butane blowtorch. **d**, Thermal conductivity of ZAGs as a function of temperature (top) and density (bottom) (steady-state thermal measurement in air). The red data highlights an ultralow *κ* of only 104 mW m^−1^ K^−1^ at 1,000 °C. **e**, Infrared radiation absorption spectra of zircon, hBN, ZrO_2_, SiO_2_ and Al_2_SiO_5_ aerogels, and asbestos. **f**, Thermal conductivity at 1,000 °C of zircon, hBN, ZrO_2_, SiO_2_ and Al_2_SiO_5_ aerogels, and asbestos. The hBN aerogel was tested at 900 °C. Error bars in **d**, **f** indicate the standard deviations of *κ*. **g**, Photograph and infrared images of ZAGs for thermal protection for the human body under a butane blowtorch. **h**, Comparison of thermal insulation performance between ZAGs and a commercial barrier (thickness about 1 cm) for an aero engine (CFM56) fuel tube under the flame of a butane blowtorch. Torch heating started at 0 min and stopped at 5 min, showing only 33 °C inside a fuel tube with a stable ZAG barrier (top) compared with 267 °C in one with a burn-down commercial barrier (bottom).

Thermal stability is another essential factor for the design of thermal insulating materials for extreme conditions. Considering the intrinsic high oxidation resistance and thermal stability of hypocrystalline zircon (Supplementary Fig. 29), we focused our discussion on *α*. The ZAGs show a near-zero *α* of 1.2 × 10^−7^ °C^−1^ below 200 °C and 1.6 × 10^−7^ °C^−1^ above 400 °C (Fig. 4a). This near-zero *α* of ZAGs could reduce the strain mismatch among fibres and prevent the well connected fibres from detaching and disintegrating (Fig. 4a, inset). The MD (Supplementary Fig. 30) and finite-element (FE) (Supplementary Fig. 2) simulations showed that the hypocrystalline zircon nanofibre exhibited a negative *α* (−11.5 × 10^−6^ °C^−1^) along the *c* direction, and an apparent positive *α* of 6.6 × 10^−7^ °C^−1^ and 1.1 × 10^−6^ °C^−1^ along the *a* and *b* directions, respectively. The negative-*α* behaviour of nanofibres, triggered by the thermally excited bending Poisson’s effect^48^ with enhancement from high-order deformation mode, induced the axial contraction, whereas the positive-*α* behaviour induced the radial expansion. As a result, the radial expansion strain (positive *α*) of fibres can be offset by the axial contraction strain (negative *α*) of connected fibres in the cell (Fig. 1a). In addition, the synergistically high-order deformation mode from the zig-zag architecture can extend the near-zero-*α* behaviour from the local to global aerogel (Supplementary Fig. 2). Conventional crystal or amorphous ceramics with randomly twined or simply laminated structures show only large positive *α*. Thus, with the multiscale design from a hypocrystalline zircon nanofibrous structure to a zig-zag architecture, our ZAGs provide an effective pathway to a near-zero *α* and greatly enhance thermal stability.

We then measured the thermal stability of the ZAGs under rapid thermal shocks and high-temperature exposures by using a home-designed pneumatic thermal-shock testing system (Supplementary Figs. 31, 32, Supplementary Video 5). The sample retained its original morphology after 10,000 thermal-shock cycles, with the ultimate stress remaining nearly unchanged and little strength degradation of <1% (Fig. 4b, inset), indicating an excellent structural stability and resistance to drastic temperature cycles. Moreover, the ZAGs did not show apparent structure collapse and strength degradation when exposed to bilateral butane blowtorch flames (over 1,300 °C, Supplementary Video 6), and retained mechanical elasticity after cyclic compressions with 50% strain under the blowtorch flames (Fig. 4c, Supplementary Fig. 33). In addition, the ZAGs showed no strength or volume loss after one-week exposure at 1,000 °C (Supplementary Fig. 34), presenting an outstanding thermal stability under high-temperature conditions. Compared with the conventional ceramic aerogels with positive or negative *α* (ref. ^19^; for example, hexagonal boron nitride (hBN), silicon carbide (SiC) Al_2_O_3_ and SiO_2_ aerogels, Supplementary Fig. 35), our near-zero-*α* material endured larger thermal-shock cycles and higher-temperature exposure in air with even less structural degradation.

We further evaluated the thermal insulating properties. The ZAGs show a low *κ* of 26 mW m^−1^ K^−1^ at 25 °C, comparable to the best values reported at room temperature. It is important to note that most ultralight ceramic aerogels reported so far show a rather high *κ* above 200 mW m^−1^ K^−1^ at high temperatures (*T*_r_) around 1,000 °C or above^6^, indicating a poor thermal insulation performance under extreme conditions. This is not surprising as the thermal radiation (*κ*_r_) scales linearly as *κ*_r_ ~ *T*_r_^3^*ρ*^−1^*e*^−1^ and becomes the dominant heat transfer path at high temperature, whereas the low density (*ρ*) and small infrared radiation absorption coefficient (*e*) of typical ceramic aerogels cannot effectively suppress the thermal radiation heat transfer^49^. To this end, the ZAGs with embedded amorphous carbon offer an interesting solution to reduce the *κ*_r_ at high temperatures by considerably increasing *e*. Indeed, compared with the typical hBN, SiO_2_, zirconium dioxide, mullite ceramic aerogels and asbestos under the same test parameters (density, thickness and porosity), the ZAGs show a more than two times as high infrared radiation absorption rate (Fig. 4e, Supplementary Fig. 36), leading to an ultralow *κ* of only 104 mW m^−1^ K^−1^ at 1,000 °C (Fig. 4d, a lower *κ* of 93 mW m^−1^ K^−1^ was achieved for a sample annealed at 800 °C owing to the higher carbon content, Supplementary Figs. 37–41, Supplementary Table 1),which represents one of the lowest *κ* values reported at 1,000 °C (Fig. 4f, Supplementary Fig. 42), demonstrating an exceptional high-temperature thermal insulating performance.

We finally evaluated the practical thermal insulating performances of the ZAGs (Fig. 4g, h). A ZAG slab with a thickness of 1.0 cm was directly placed on the hand and a butane blowtorch flame (about 1,300 °C) was used to heat the top surface (Fig. 4g). After heating for 5 min, the temperature at the slab bottom maintained a relatively low temperature of only 37 °C, within the tolerance of the human body. Moreover, we tested the ZAGs as a typical thermal barrier for the aero engine (CFM56) fuel tubes (Fig. 4h). After heating with a butane blowtorch flame for 5 min, the temperature of the inner fuel tube with a commercial polyimide foam barrier and conventional SiO_2_ fibrous aerogel barrier (Supplementary Fig. 43) increased to 267.2 °C and 159.1 °C, respectively, whereas that with the ZAG barrier remained below 33 °C (Supplementary Video 7). To further mimic the complex working environments under extreme conditions, including the typical mechanical vibrations under high temperatures, we investigated the thermal insulating performance of ZAGs under 400 °C with a vibration amplitude up to 30% of the sample thickness at 0.25 Hz (Supplementary Fig. 44, Supplementary Video 8). The thermal profiles of the ZAGs showed the temperature at the top area remained below 40 °C when the sample was heated from the bottom at 400 °C under 100-times vibration. In addition, the ZAGs exhibit a low permittivity relative to air of about only 1.437 (Supplementary Fig. 45), and can be used for a capacitive strain sensor for condition monitoring (Supplementary Figs. 46, 47). This self-sensing thermal insulation material can further ensure its operational safety by real-time monitoring the structural cracks and strength degradation.

We have shown that a multiscale design of hypocrystalline zircon nanofibrous aerogels with a zig-zag architecture produces a thermomechanical metamaterial with near-zero Poisson’s ratio and near-zero thermal expansion coefficient. The resulting material features a unique combination of superior thermomechanical stability and high-temperature thermal insulating performances beyond the reach of the typical ceramic aerogels, thus defining a robust material system for thermal insulation under extreme conditions, such as spacecrafts, lunar and Mars bases, deep-earth detectors, furnaces, and space and fire suits. Moreover, the double-near-zero-index aerogels also provide opportunities for thermal management of strain-sensitive electronic devices, optical devices and superconductive devices.

## Methods

### Sample synthesis

Ceramic nanofibrous aerogels were prepared using a turbulent-flow-assisted electrospinning (TFAE) method. On the basis of a typical fabrication of polyacetylacetonatozirconium (PAZ)^50^, we developed a stepwise dissolution process to synthesize the electrospinning precursor. First, the zirconium–silicon precursor sol, was produced from a mixture with a molar composition of PAZ:yttrium nitrate hexahydrate:triethoxysilane:solvent (methanol, ethanol or water) = (about 1.50–1.75):0.095:(about 0.77–0.92):(about 0.65–0.85). The mixture was stirred for 1 h to obtain a clear and golden-transparent solution. Then, polyethylene oxide (PEO, weight-averaged molecular mass *M*_w_ = 1,000000) was added to the zirconium–silicon precursor sol with a sol:PEO weight ratio of 1,250:1 at a temperature of 60 °C, and stirred for 80 min to obtain the eventual electrospinning solution. After defoaming, the resulting solution was used directly for TFAE to obtain the as-spun ceramic nanofibres. We developed a coaxial TFAE system. Forty millilitres of acquired solution was fed into plastic syringes fixed in a microsyringe pump, and the spinning solution was injected through the 28G-nozzle spinneret with a speed of 2 ml h^−1^. The electrified liquid droplet was stretched and elongated to nanofibres from the spinneret via the direct-current, constant-high-voltage (25 kV) power and the coaxially high-speed air flow (airflow velocity of 15 m s^−1^), generating the ceramic nanofibres (Supplementary Figs. 5, 6). The as-spun nanofibres were collected on the aluminium collector at a distance (about 15–20 cm) from the nozzle. Then, we prepared the as-spun ceramic nanofibres with a zig-zag architecture by a controllable mechanical folding process (Supplementary Fig. 7). The resulting 3D zig-zag ZAGs were sintered to pre-crystallization in a Muffle furnace under flowing air to form the hypocrystalline ZAGs. The sintering process can be divided into two stages: slow heating at 2 °C min^−1^ from room temperature to 1,100 °C; and maintaining at 1,100 °C for 60 min. Next, with a secondary sintering treatment (same as above conditions) to crosslink the ZAG units, we fabricated the well assembled ZAGs with a controllable density (about 15–55 mg cm^−3^) and diverse shapes (Supplementary Fig. 7). Except as noted, all of the material and physical property investigations were performed using a sample with a density of 20 mg cm^−3^. In addition, we fabricated the typical SiO_2_, zirconium dioxide and mullite nanofibrous aerogels using the same TFAE method, and the hBN aerogels were prepared through the 3D graphene template-assisted chemical vapor deposition method^19^.

### Material characterization

The morphology was investigated by SEM and TEM on a ZEISS, Merlin Compact at 20 kV and a FEI, Tecnai G^2^ F30 with EDS at 300 kV. The elemental and structural analyses were carried out by XPS, XRD and Raman spectroscopy on a ThermoFisher, ESCALAB 250Xi, a Panalytical, X’Pert and a Renishaw, inVia-Reflex with a 532-nm laser. The mechanical properties of the ceramic aerogels were studied using an Instron 3365 universal testing machine with 100-N load cells at a loading rate of 2 mm min^−1^. The weight of the samples was measured by a Sartorius analytical balance (BSA124S-CW) with an accuracy of 0.1 mg. The thermal-shock tests of the ceramic aerogels were carried on a homemade pneumatic system with a cold source (25 °C) and a hot source (1,000 °C). The thermal expansion coefficient of the ceramic aerogels was investigated using a Thermal dilatometer (L75VD1600LT, Linses). The thermal conductivity of the ceramic aerogels was measured by a steady-state thermal conductivity tester (DRPL-V, Xiangtan Xiangyi Instruments, heater at 75−1,350 °C and chiller at 20 °C in air). The infrared absorption spectra in the 0.25–2.5 μm range were recorded using an ultraviolet–visible near-infrared spectrometer (Lambda 950) with an integrating sphere unit and automated reflectance measurement unit (sample size of 30 × 30 × 1 mm^3^ and density of 20 mg cm^−3^). The infrared images of ZAGs were recorded using a Flir A615 (−40 °C to 2,000 °C) infrared thermal camera on an optical platform. The capacitance change was measured by a Precision LCR digital bridge (VC4092A).

### MD simulation

#### Design of hypocrystalline ceramics

We investigated the physical properties of crystal, amorphous and hypocrystalline ceramics at different scales by MD simulations. First, the *ν* of three types of bulk sample with 2,304 atoms were calculated by using the Voigt−Ruess−Hill^51^ formalism at 25 °C. To be specific, a crystal sample with a size of 2.64 × 2.64 × 3.59 nm^3^ was duplicated from a 24-atom unit cell. An amorphous sample with a size of 3.17 × 3.18 × 3.15 nm^3^ was generated by melting the crystal sample at 5,000 °C for 100 ps and then quenching to 25 °C at a cooling rate of 1 °C ps^−1^. A hypocrystalline sample with a size of 2.81 × 3.36 × 3.21 nm^3^ was generated by duplicating a 576-atom unit cell of amorphous and crystal ceramics. Second, we performed thermal expansion simulations for short fibres of three different phases with a slenderness ratio of about 5, as shown in Fig. 1a. To be specific, a crystal sample with a size of 11.55 × 1.99 × 1.98 nm^3^ containing 3,888 atoms, an amorphous sample with a size of 10.91 × 2.11 × 1.94 nm^3^ containing 3,456 atoms, and a hypocrystalline sample with a size of 11.23 × 2.06 × 2.1 nm^3^ containing 3,316 atoms were used for the thermal expansion simulations. Starting from 25 °C, the temperature was increased to 1,300 °C at a heating rate of 5 °C ps^−1^ and then equilibrated at 1,300 °C for 200 ps. A time step of 1 fs was used for the integration of the motion equations.

We further investigated the values of *ν* and *α* in the crystal, amorphous and hypocrystalline cells consisting of four crosslinked long fibres, as shown in Fig. 1a. Fibres of three different phases with a slenderness ratio of about eight were first generated, and each fibrous sample was then replicated and rotated into four crosslinked fibres, which together made up the square cells. Specifically, four crosslinked fibres made up a crystal cell with a size of 16.4 × 1.99 × 16.4 nm^3^ containing 18,792 atoms, an amorphous cell with a size of 16.58 × 2.11 × 16.58 nm^3^ containing 17,984 atoms, and a hypocrystalline cell with a size of 16.67 × 2.06 × 16.67 nm^3^ containing 17,398 atoms. The thermal expansion simulations were performed the same as those with the short fibres mentioned above. To perform the compression simulations, we fixed the atoms at the two ends of the cells in the longitudinal direction and applied the displacement over the fixed atoms with the compression rate of 1 × 10^9^ s^−1^.

We increased the slenderness ratio of the fibres to about 20 and generated larger square cells with crystal, amorphous and hypocrystalline ceramics with various crystal:amorphous volume ratios. To be specific, four crosslinked fibres made up a crystal cell with a size of 45.0 × 1.99 × 45.0 nm^3^ containing 53,312 atoms, an amorphous cell with a size of 45.0 × 2.11 × 45.0 nm^3^ containing 54,038 atoms, a hypocrystalline cell (crystal:amorphous volume ratio of 1:1) with a size of 45.0 × 2.06 × 45.0 nm^3^ containing 50,514 atoms, a hypocrystalline cell (crystal:amorphous volume ratio of 1:2) with a size of 45.0 × 2.06 × 45.0 nm^3^ containing 50,289 atoms, and a hypocrystalline cell (crystal:amorphous volume ratio of 2:1) with a size of 45.0 × 2.06 × 45.0 nm^3^ containing 49,833 atoms (Supplementary Fig. 1a). We performed the compression and thermal expansion simulations in triangle, pentagon and hexagon cells with a crystal:amorphous volume ratio of 1:1. A triangle cell with a size of 45.0 × 2.06 × 39.79 nm^3^ containing 37,603 atoms, a pentagon cell with a size of 67.04 × 2.06 × 62.9 nm^3^ containing 62,848 atoms, and a hexagon cell with a size of 78.16 × 2.06 × 71.14 nm^3^ containing 75,165 atoms were used for the calculation (Supplementary Fig. 1b).

#### Crystallization simulations

We performed the WTMetaD simulations for 100 ns under temperatures ranging from 1,300 °C to 1,700 °C. To distinguish the states of tetragonal zirconium dioxide and amorphous zircon, we chose two different collective variables with the number of bridging oxygens as ${s}_{1}$ and the dot product of the local Steinhardt’s order parameter^52–54^ ${q}_{4}^{{\rm{dot}}}$ of zirconium as ${s}_{2}$. We started the WTMetaD simulations with 576 atoms of amorphous zircon with a molar ratio of Zr:Si:O = 2:1:6. The isothermal–isobaric MD simulations was performed using a Nose–Hoover thermostat and barostat^55,56^. The short-range interaction for WTMetaD simulations was described by the Buckingham form potential^57^ with a cut-off distance of 8 Å. For the long-range Coulombic term, the cut-off was set to 10 Å with the Ewald sum method. The bias factor $\gamma$ of the WTMetaD was set to 50. The Gaussians with a height of 40 kJ mol^−1^ and width of 2 and 0.04 collective variable units for ${s}_{1}$ and ${s}_{2}$ were introduced to construct the bias potential of the WTMetaD. All the simulations were carried out with the Large-scale Atomic/Molecular Massively Parallel Simulator (LAMMPS)^58^ code, and the WTMetaD simulations were carried out with an additional plugin code^59^ PLUMED 2. We constructed the model of complete hypocrystalline zircon by adding carbon atoms to the system obtained from WTMetaD at 1,700 °C, which showed the highest crystallinity from the local-entropy-based fingerprint^60,61^ $\bar{{\rm{s}}}$. We performed the rest of the MD simulations of hypocrystalline zircon using the ReaxFF reactive force field^62,63^. The ReaxFF force field was constructed by combining the parameters of the C/Zr/O force field^64^ and the parameters of the C/Si/O force field^65^.

#### Mechanical properties

We duplicated the system from a 576-atom cell into a 2.67 × 3.25 × 3.47 nm^3^ sample with 2,304 atoms and replaced atoms inside a spherical region (radius of 0.2 nm) in amorphous zircon part with 20 randomly distributed carbon atoms. The 2,315-atom sample was further duplicated to a 2.67 × 3.25 × 111.12 nm^3^ fibrous sample with 74,080 atoms. We first performed bending simulation of the 74,080-atom fibrous sample with slenderness ratio of about 30. The fibre was compressed to the maximum strain of 60% and recovered to its original length. We then performed fracture simulation of the fibre sample along the *c* coordinate axis. The tensile strength was obtained by increasing the box size ${L}_{{\rm{c}}}$ of the system stepwise by 0.1% of the initial value ${L}_{{\rm{c}}0}$ until the fracture of the fibre. The tensile stress along the *c* axis was then computed using the virial theorem^66^.

#### Thermal properties

We first performed *α* simulation for the 74,080-atom fibrous sample and calculated the *α* by MD simulation. The temperature was increased stepwise by 50 °C from 0 °C to 400 °C at a heating rate of 5 °C ps^−1^, and relaxed at each step for 20 ps to obtain a statistical average of *α*. We then performed two sets of non-equilibrium MD simulations and calculated *κ* using a direct method to show the reduction of *κ* in hypocrystalline zircon caused by the atomic arrangement and the crosslinking pattern. We calculated *κ* of bulk single-crystal zircon and the hypocrystalline zircon samples. To be specific, for crystal zircon, we duplicated the unit cell of zircon into two 6,144-atom samples with a size of 10.57 × 2.64 × 2.39 nm^3^ and 2.64 × 2.64 × 9.56 nm^3^ for the calculation of *κ* along the *a* and *c* directions. For hypocrystalline zircon, we replaced atoms inside a spherical region (radius of 0.2 nm) in the amorphous zircon part (576 atoms) with 20 randomly distributed carbon atoms, and then duplicated the hypocrystalline zircon to three samples with sizes of 10.42 × 2.67 × 3.25 nm^3^, 3.47 × 10.68 × 1.62 nm^3^ and 3.47 × 2.67 × 9.72 nm^3^ to obtain *κ* along the *a*, *b* and *c* directions. Hot and cold regions were set vertical with the longitudinal direction and located at two sides of the system with a thickness of 2 Å. Furthermore, we calculated *κ* of two nanofibrous samples overlapping vertically, compared with samples placed side by side. We used two 14,088-atom fibrous systems with the same size of 2.67 × 3.25 × 20.84 nm^3^ duplicated from a 587-atom hypocrystalline system. Hot and cold regions were set at the middle of each fibre along the longitudinal axis with a thickness of 2 Å.

#### Carbon retainment

The reactive MD simulations were performed in a canonical ensemble, and a Berendsen thermostat^67^ was used to control the temperature of the system. Periodic conditions were used along each boundary of simulation box. A time step of 0.25 fs was used for the integration of the motion equations. First, we generated a sample containing 288 randomly distributed atoms with a molar ratio of Zr:Si:O = 1:2:6 and melted the sample at 5,000 °C for 100 ps to ensure that the atoms were fully relaxed. The sample was then quenched to 25 °C at a cooling rate of 1 °C ps^−1^. The sample was duplicated to cubes with different lengths of 2 nm, 3 nm and 4 nm. The zircon atoms inside a cylinder region in the centre of the cubes with radius of 0.5 nm were replaced with 50, 75 and 100 randomly distributed carbon atoms, respectively. Furthermore, 100, 225 and 400 oxygen atoms were placed randomly in the oxygen regions (space of 2 nm above and below the amorphous zircon region) in the three systems, respectively. In contrast, for the hypocrystalline zircon sample, we generated a quarter of a cylinder of tetragonal zirconium dioxide with a radius of 1.5 nm at each corner of the cubic amorphous zircon. We performed 10 ps of simulation at a temperature of 25 °C for each system and then sintered at a temperature of 1,100 °C for 500 ps.

Additional details of the MD simulation can be found in the Supplementary Information.

### FE simulation

In the FE simulations, we designed a zig-zag architecture in the aerogel to trigger an additional high-order deformation at the macroscale to mitigate the structure-derived increase of *ν* and *α* to achieve the near-zero responses (Fig. 1b, Supplementary Fig. 2). In geometric morphologies, a 2D porous zig-zag architecture was constructed by laminated fibrous layers and corner joints with high-order deformation fibrous cell as building blocks. Three shapes of cells (triangle, pentagon and hexagon) were used to assemble the fibrous aerogel with zig-zag architectures. To simplify the simulation process, a 2D FE model was established and the characteristics of fibrous units were simulated using a beam element with the geometric parameter set as 13:1 for the slenderness ratio. For *ν*, compression loadings were added by a displacement of the top layer at 5% strain with the model fixed at the bottom. For *α*, the temperature was gradually increased from 0 °C to 400 °C to investigate the thermally excited response. The material attributes were set similar to those of the hypocrystalline ceramics from MD simulations.

### Computational fluid dynamics simulation

In the numerical simulation, a large eddy simulation with kinetic-energy transport subgrid-scale model was adopted to solve the Navier–Stokes equation, which obtains 3D unsteady flow motions. The computational domain, boundary conditions and mesh are shown in Supplementary Fig. 3. The coaxially circular nozzle was 30 mm in length with an inner diameter of 5 mm. The dimension of the collector was 560 × 480 × 450 mm^3^. The pressure inlet with a gauge total pressure of 10 kPa was assigned to the coaxially circular nozzle, and no-slip boundary conditions were used for the other surfaces. The simulations were carried out using commercial software Fluent Solver 2020 R1. The finite-volume method was used in the solver, and the pressure–velocity coupling was achieved with the pressure implicit with the splitting of operators method. Least-squares cell-based, bounded central differencing and second-order upwind schemes were adopted to discretize the gradient, momentum and subgrid kinetic energy terms, respectively. The second-order implicit scheme was adopted for the temporal discretization. The time step was adaptive with a courant number of 1. Supplementary Fig. 4 presents the instantaneous turbulent structures and fibrous streamlines of the 3D turbulent flow. The turbulent structures were obtained by the Lambda2 (*λ*_2_) criterion.

## Online content

Any methods, additional references, Nature Research reporting summaries, source data, extended data, supplementary information, acknowledgements, peer review information; details of author contributions and competing interests; and statements of data and code availability are available at 10.1038/s41586-022-04784-0.

## Supplementary information

Supplementary Information This file contains Supplementary Figs. 1–47, Supplementary Table 1, legends for Supplementary Videos 1–8 and Supplementary References.

Supplementary Video 1

Supplementary Video 2

Supplementary Video 3

Supplementary Video 4

Supplementary Video 5

Supplementary Video 6

Supplementary Video 7

Supplementary Video 8

## Data Availability

All data to support the findings of this study are present in the paper and its Supplementary Information. Raw data are available from the corresponding authors on reasonable request.
