# Supplementary material for: Hypocrystalline ceramic aerogels for thermal insulation at extreme conditions
Source: Nature. 2022 Jun 29;606(7916):909–16. doi: 10.1038/s41586-022-04784-0 (PMC9242853; doi:10.1038/s41586-022-04784-0)
Supplement: Supplementary file 1 — This file contains Supplementary Figs. 1–47, Supplementary Table 1, legends for Supplementary Videos 1–8 and Supplementary References. [file 41586_2022_4784_MOESM1_ESM.pdf]

nature portfolio

https://doi.org/10.1038/s41586-022-04784-0

Supplementary information

Hypocrystalline ceramic aerogels for thermal insulation at extreme conditions

In the format provided by the authors and unedited

Supplementary Information

Hypocrystalline ceramic aerogels for thermal insulation at extreme conditions

Jingran Guo[1]*, Shubin Fu[1]*, Yuanpeng Deng[1]*, Xiang Xu[1]†, Shujin Laima[1], Dizhou Liu[1], Pengyu Zhang[1], Jian Zhou[1], Han Zhao[1], Hongxuan Yu[1], Shixuan Dang[1], Jianing Zhang[1], Yingde Zhao[1], Hui Li[1]†, and Xiangfeng Duan[2]†

[1]Key Lab of Smart Prevention and Mitigation of Civil Engineering Disasters of the Ministry of Industry and Information Technology and Key Lab of Structures Dynamic Behavior and Control of the Ministry of Education, Harbin Institute of Technology, Harbin, 150090, China; [2]Department of Chemistry and Biochemistry, University of California, Los Angeles, California 90095, USA

†Correspondence to: xuxiang@hit.edu.cn, lihui@hit.edu.cn, xduan@chem.ucla.edu

This file includes:

Supplementary Materials

Supplementary Fig. 1-47

Supplementary Table 1

Captions for Supplementary Video 1-8

References

Other Supplementary Material for this manuscript includes the following:

Supplementary Video 1-8 (.mp4)

Additional details of MD simulation

Crystal/amorphous/hypocrystalline ceramic nanofibers

The double-near-zero metaproperty of ceramic nanofibrous aerogels is mainly attributed to the high-order deformation mode of hypocrystalline ceramics and Zig-Zag architecture design. Three kinds of phase ceramics (crystal, amorphous and hypocrystalline (Fig. 1a)), were first investigated to discuss the impact of material structure. From the MD simulations, crystal ceramics show long-range order with periodical arrangement of atoms, while amorphous ones only exhibit short-range order at the atomic length scale and isotropic properties in larger scale. However, hypocrystalline ceramics show the medium-range order comparing to crystal and amorphous ones. With inhomogeneous distributed nanocrystals embedded in amorphous matrix, the hypocrystalline ceramics show remarkable anisotropy within nanometer scale. The anisotropic properties caused by asymmetry can trigger high-order deformation mode under thermal or mechanical excitations, resulting in the double-near-zero metaproperty of hypocrystalline cells. We here illustrate the physical properties of hypocrystalline fibers by MD simulations at different scales.

Firstly, the v of crystal/amorphous/hypocrystalline samples with 2304 atoms were calculated at 25 °C. The crystal and amorphous ceramic samples show v of 0.302 and 0.29, respectively, while the hypocrystalline sample exhibits lower v of 0.277. The reduction of v in bulk hypocrystalline system is basically attributed to compatible deformation of the components in the hypocrystalline ceramics. Secondly, we performed thermal expansion simulations for short fibers of three different phases. As demonstrated in Fig. 1a, the crystal ceramic shows little deformation, the amorphous one shows 1st order deformation mode, while the hypocrystalline one shows tendency of high-order deformation mode after thermal treatment. Thirdly, we validate the near-zero values of v and α in crystal/amorphous/hypocrystalline cells based on square shape, which consisted of 4 crosslinked long fibers. As shown in Fig. 1a, amorphous fibers would show 1st order deformation both under compression and thermal treatment, while crystal fibers would keep straight under thermal treatment, leading to higher α_c than α_a. For the compression simulation, densification process would occur before bending deformation in amorphous cell, resulting in lower v_a than v_c under same compressive strain. Different from crystal and amorphous samples, the design of hypocrystalline cell would increase degrees of freedom and promote the high-order deformation mode under thermal and mechanical excitations, resulting in near-zero values of v and α.

To better understand the size effect in fibrous aerogel and the impact of volume ratio in hypocrystalline ceramic, we generated five larger square samples (Supplementary Fig. 1a) with crystal, amorphous, and three hypocrystalline samples with crystal/amorphous volume ratio of 2:1, 1:1, and 1:2. We performed the compression and thermal expansion simulations for these five cells. As can be seen in Supplementary Fig. 1a, with the increase of slenderness ratio, all hypocrystalline cells exhibit higher order deformation mode than that in Fig. 1a, while crystal and amorphous cells would remain 1st order deformation. Among three types of hypocrystalline cells with different crystal/amorphous volume ratio, the hypocrystalline cell with equal proportion of crystal and amorphous part shows the strongest tendency in high-order deformation. To illustrate the double-

near-zero metaproperty of hypocrystalline cells occur in different geometrical patterns, we further performed the compression and thermal expansion simulations with triangle, pentagon, and hexagon cells, which constructed by hypocrystalline fibers with crystal/amorphous volume ratio of 1:1. As shown in Supplementary Fig. 1b, the different geometrical hypocrystalline cells also present a high-order deformation mode under thermal and mechanical excitations.

Mechanism of the formation of tetragonal zirconia

In accordance with the XRD patterns (Fig. 2c) of the nanofiber, we found that the as-spun nanofibers crystallized into the tetragonal zirconia at above 1000 °C. Under a vacuum atmosphere, 3 mol% of Y_2O_3 would increase the formation of t-ZrO_2, and further prompt zircon synthesis[1,2]. The same reaction conducted under an air atmosphere would result in different reaction products. Yttrium stabilized t-ZrO_2 has very high oxygen conductivity due to the oxygen vacancies caused by doping. Under the oxygen-enriched environment of air atmosphere, oxygen would enter the lattice of yttrium stabilized t-ZrO_2 easily under high temperature sintering. The absorbing of oxygen would then increase the activation energy of silicon migration into the lattice of t-ZrO_2, and therefore significantly reduced the synthesis of zircon[3]. For zircon with Si/Zr molar ratio of 1 and stabilized with 3 mol% of Y_2O_3, only 5 wt.% of zircon in the crystalline phase will form under an air atmosphere sintered in 1400 °C for 6 h[3]. In addition, for the case of unstabilized zircon (i.e. without yttrium doping), the formation of zircon is strongly affected by the molar ratio of Si/Zr[4]. Zircon phase would hardly synthesis[4] with the Si/Zr molar ratio lower than 0.25. With the increase of the Si/Zr molar ratio, the formation efficiency of zircon phase would increase[5] and reach the top at molar ratio of 1.2. Therefore, the absorbing of oxygen in the lattice of yttrium stabilized t-ZrO_2 and the reduction of Si/Zr molar ratio to 0.5 would together lead to the synthesis and stabilize of t-ZrO_2 in nanofibers of current work.

MD simulations of hypocrystalline zircon

a) Metadynamics for crystallization simulation

To better understand the crystallization process of single fiber under sintering, we performed well-tempered metadynamics (WTMetaD)[6] simulation for 100 ns under the temperature from 1300 °C to 1700 °C with an interval of 100 °C. WTMetaD can help the system overcome energy barriers by introducing a history dependent bias potential which depends on a set of well-chosen collective variables (CVs). The CVs should be able to distinguish between different states of the simulated system and be functions of the coordinates of the atoms. After a long enough simulation, the probability distribution of CVs (s) becomes

$$P(\boldsymbol{s}, t \to \infty)d\boldsymbol{s} \propto e^{-[F(s)/T+\Delta T]}d\boldsymbol{s} \quad (1)$$

where T is the system temperature, ΔT is a temperature which is calculated from a preselected bias factor $\gamma = (T + \Delta T)/T$. The bias factor γ is introduced to prevent the system falling into regions with much higher energy which are not physically relevant. When WTMetaD simulation reach convergence, a more frequent $\boldsymbol{s}$ indicates the representing state is of lower free energy, while CV value that didn't appear indicates the free energy of the representing state is out of

consideration. We recommend the readers ref.[6] for more theoretical information about WTMetaD simulation.

To simplify the issues, we ignore the yttrium doping and focus on crystallization process from amorphous zircon to that with crystal phase of tetragonal zirconia. The amorphous carbon atoms were neglected in metadynamics simulation since they didn't contribute to the crystallization process. We considered the crystallization process is two steps, one is the zirconium and silicon ions getting separated and forming zirconium-rich and silicon-rich regions, and the other is the crystallization process of tetragonal zirconia. In order to distinguish the states of tetragonal zirconia and amorphous zircon, we chose two different CVs, the number of bridging oxygen (BO) as s_1, and the dot product of local Steinhardt's order parameter[7-9] q_4^{dot} of zirconium as s_2. We were inspired by ref.[10] to use the number of BO to characterize the enrichment degree of silicon ions. From our system with the Si/Zr molar ratio of 0.5, the more BO indicates that there are more silicon-rich regions, while the lower BO indicates the zirconium and silicon ions are mixed more uniformly. By using BO as a CV, we can perform the first step of the crystallization process (i.e. the separation of zirconium and silicon ions).

The dot product of local Steinhardt's order parameter q_4^{dot} of zirconium can distinguish between crystal and amorphous states. We first calculate the Steinhardt's order parameter q_l of zirconium with oxygen ions:

$$q_l(i) = \sqrt{\frac{4\pi}{2l+1}\sum_{m=-l}^{l}\left|\frac{1}{N_i}\sum_{j\in\Omega_i} f_1(r_{ij})\, Y_{lm}(\hat{r}_{ij})\right|^2} \tag{2}$$

where $\hat{r}_{ij} = (\vec{r}_i - \vec{r}_j)/|\vec{r}_i - \vec{r}_j|$ is the unit vector connects the atoms i and j, Y_{lm} is the spherical harmonics, Ω_i is the coordination sphere around atom i. A cubic cutoff function $f_1(r_{ij}) = \left(\frac{r_{ij}-r_1}{r_0-r_1} - 1\right)^2\left[1 + 2\left(\frac{r_{ij}-r_1}{r_0-r_1}\right)\right]$ is introduced to smooth the boundary of Ω_i, where r_0 is set to 3 Å and r_1 is set to 3.1 Å, while for $r_{ij} \le r_0$ the cutoff function is equal to unity. The parameter q_l can characterize the degree of order of the neighbor atom j (oxygen) in the coordination sphere around center atom i (zirconium). We can then give a vector form of $q_l(i)$:

$$\boldsymbol{q}_l(i) = (q_{l,l}, q_{l,l-1}, \ldots, q_{l,-l+1}, q_{l,-l}) \tag{3}$$

and the dot product of $\boldsymbol{q}_l$, q_l^{dot} can be given as follow:

$$q_l^{\mathrm{dot}}(i) = \frac{\sum_j f_2(r_{ij})\boldsymbol{q}_l(i)\cdot\boldsymbol{q}^*{}_l(j)}{\sum_j f_2(r_{ij})} \tag{4}$$

where i and j are both zirconium atoms, $f_2(r_{ij})$ is another cubic cutoff function, and the r_0 is set to 4.5 Å and r_1 is set to 4.6 Å according to the first peak of pair distribution function $g_{\mathrm{Zr-Zr}}(r)$. The parameter q_l^{dot} can tell whether the ordered zirconium atoms are clustered together or not. It can be seen in Supplementary Fig. 13 that q_4^{dot} can distinguish between crystal phase of tetragonal zirconia and amorphous zircon very well. With the WTMetaD simulation, the two CVs we chose can drive the system from amorphous zircon to the hypocrystalline systems of amorphous zircon

with tetragonal zirconia.

We started the WTMetaD simulation with 576 atoms of zircon with a molar ratio of Zr:Si:O=2:1:6, and atoms were randomly distributed in the simulation box. It can be found that at the temperature of 1300 °C, the system remained disordered after 100 ns of WTMetaD simulation, while the crystallization process occurred from the temperature of 1400 °C to 1700 °C. The system would turn into a mixture of tetragonal zirconia and amorphous zircon in the WTMetaD simulation from 1400 °C to 1700 °C. At the temperature of 1400 °C and 1500 °C, the system would crystallize and retained crystalline during 100 ns simulation, indicating that at temperature below 1500 °C the free energy of crystal state is lower than the amorphous one under the potential we used. For the case of 1600 °C and 1700 °C, more phase transition process occurred between crystalline and non-crystalline structure. The snapshots of crystalline system obtained at the temperature of 1400 °C to 1700 °C, and the snapshot of amorphous structure at 1300 °C are listed in Supplementary Fig. 14. To characterize the crystallinity of the hypocrystalline zircon obtained at each temperature, we calculated the local entropy based fingerprint $\bar{s}$ of the zirconium atoms according to ref.[11,12]. A higher $\bar{s}$ value indicates the zirconium atom is more liquid-like, while a lower $\bar{s}$ value refers to more crystal-like zirconium atom. It can be found that with the increase of temperature, the hypocrystalline zircon obtained by WTMetaD would show higher crystallinity. We further calculated the XRD intensity of these crystalline structures (amorphous structure at 1300 °C as a contrast) obtained with WTMetaD by Debye scattering function[13] and the result shows good match with the experimental result as shown in Supplementary Fig. 14.

The results show that the CVs we chose can characterize the difference between amorphous zircon and that with tetragonal zirconia, and can achieve the crystallization process to be the same as the reaction experimentally. We further constructed the model of complete hypocrystalline zircon by adding the carbon atoms to the system with tetragonal zirconia and amorphous zircon obtained from WTMetaD at 1700 °C, and performed the rest MD simulations with the complete hypocrystalline zircon samples by using ReaxFF reactive force-field[14,15].

b) Bending simulation

To better show the deformability of single nanofiber, we performed bending simulation of fibrous sample constructed by the system obtained from WTMetaD simulation. It should not be ignored that the nanofiber has much larger slenderness ratio than bulk materials. The carbon atoms inside the amorphous zircon may also influence mechanical and other properties. In order to be closer to the real conditions in experiment, we generated a 74080 atoms fibrous sample with slenderness ratio of ~30. The fiber was compressed to the maximum strain of 60% with compression rate of 1×10^9 s^{-1}. After the compression simulation, we performed tension simulation to straighten the bended system to its initial length. It can be seen in Supplementary Fig. 24 that the fibrous system recovered to its original state without significant breakage. The bending deformability of single nanofiber can be well proved by the simulation results.

c) Fracture simulation

To evaluate the mechanical properties of single nanofiber, we performed fracture simulation and calculated the tensile strength of the fibrous system with 74080 atoms along the c coordinate axis. As shown in Fig. 3g, the nanofiber had tensile Young's modulus of 173.5 GPa and fractured at 6.7% strain with tensile strength of 7.9 GPa. It can be seen in Supplementary Fig. 25 that the fracture occurred at the surface between tetragonal zirconia and amorphous zircon. It should be noted that the model is defect-free and with slenderness ratio much lower than the nanofiber in experiments, and thus it is only an ideal model. We need to say that compared with the simulation results, the mechanical properties of nanofibers in experiments with defect and higher slenderness ratio will be lower.

d) Thermal expansion simulation

In section above, we have discussed the near-zero α property of hypocrystalline cell. Here we performed thermal expansion simulations for nanofibers with a higher slenderness ratio (30:1) and calculated the α by MD simulations from 0 °C to 400 °C. We chose the fibrous sample with 74080 atoms to show the influence of longitudinal size on the α of nanofibers. The α result along a, b and c direction and the snapshots of the longer fiber model at 0, 200 and 400 °C are shown in Supplementary Fig. 30. The radial positive α along a and b direction maintained lower than 2×10^{-6} °C^{-1}, which is much lower than the negative α along c direction of about 11.5×10^{-6} °C^{-1}.

e) Reaction of carbon oxidation embedded in hypocrystalline zircon

We performed reactive molecular dynamics simulation to verify the effectiveness of hypocrystalline zircon served as the oxygen diffusion barrier layer and prevent inner carbon from oxidation. Firstly, we tested the oxygen diffusion barrier effect of amorphous zircon without tetragonal zirconia nanocrystals. Considering that the vast majority of zirconium atoms are concentrated in polycrystalline zirconia and carbon atoms mainly distributed in amorphous zircon, we used a sample of amorphous zircon containing 288 atoms with Zr/Si molar ratio of 0.5. To verify the thickness dependence of amorphous zircon served as the oxygen diffusion barrier layer, we generated three different systems with protection layer thicknesses of 0.5 nm, 1nm, and 1.5 nm, sheltering the internal carbon cylinder. As shown in Supplementary Fig. 16, all the systems show insufficient protective effect preventing carbon from oxidation after 500 ps of sintering. To be specific, about half of carbon atoms were oxidized in the system with zircon layer of 0.5 nm, and a few carbon atoms were oxidized in the system with zircon layer of 1 nm. For the system with zircon layer of 1.5 nm, there was still one carbon atom oxidized. The result indicated that the increase of thickness of amorphous zircon layer could improve the protection by preventing carbon from oxidation, but only to a limited extent. Thus, using amorphous zircon solely as oxygen diffusion barrier layer was not able to completely prevent the migration and oxidation of carbon atoms.

We further performed simulations to verify the contribution of crystalline tetragonal zirconia for the stabilization of carbon atoms. To better match with experimental structure in nanofibers, each corner of the cubic sample of amorphous zircon was replaced with a quarter of a cylinder tetragonal

zirconia as shown in Supplementary Fig. 16. Carbon atoms remained stable in the middle part of the system within 500 ps of simulation. The inner amorphous zircon layer was restricted by zirconia nanocrystals due to the pinning effect, and served better as oxygen diffusion barrier layer, leading to more stable state of carbon atoms. Combining the amorphous and crystal part, the hypocrystalline zircon can better protect internal carbon from oxidation under high temperature of ~1100 °C and therefore increase the carbon content in the aerogels.

f) Interface between amorphous carbon and hypocrystalline zircon

We further examined the impact of interface between amorphous carbon and hypocrystalline zircon under thermal and mechanical excitations. To better show the contact between carbon atoms with amorphous zircon, we chose the hypocrystalline sample as discussed in section e) which has higher carbon proportion than that in experiment. Thermal expansion and tensile fracture simulations were performed to show the impact of interface. As shown in Supplementary Fig. 17, the region formed by carbon atoms would deform and the interface between carbon and hypocrystalline zircon would blend together under thermal treatment. Some carbon atoms may migrate into amorphous zircon at the temperature of 400 °C. Tensile fracture tends to occur in the carbon region and crack would then grow along interface between amorphous zircon and tetragonal zirconia. As a result, the thermal and mechanical properties of fibrous aerogel can be affected by the embedded amorphous carbon at their interface.

g) Thermal conductivity simulation

We performed two sets of MD simulations and calculated the thermal conductivity by using direct method to show the reduction of thermal conductivity in hypocrystalline zircon caused by atomic arrangement and crosslinking pattern. The thermal conductivity is calculated in a non-equilibrium molecular dynamics (NEMD) simulation using the Fourier law,

$$k = -\frac{q}{\partial T / \partial z} \tag{5}$$

where q is the heat flux, ∂z is the distance between the hot and cold regions, and $\partial T / \partial z$ is the temperature gradient between the hot and cold regions.

To better understand the changes of thermal conductivity between the single-crystal zircon and the hypocrystalline zircon, we first calculated the thermal conductivity of these two configurations in bulk system. To reduce the length dependences of thermal conductivity in direct method[16], we used the crystal zircon and hypocrystalline zircon with nearly the same longitudinal length. For single-crystal zircon, thermal conductivity along a and c direction were calculated, while for hypocrystalline zircon, thermal conductivity along a, b and c direction were calculated (Supplementary Fig. 37). The average thermal conductivity of crystal zircon shows to be 1.35 W m^{-1} K^{-1}, while the average thermal conductivity of hypocrystalline zircon shows to be 0.55 W m^{-1} K^{-1} (Supplementary Fig. 39), indicating the reduction of thermal conductivity caused by the atomic arrangement of the hypocrystalline system. Furthermore, we used direct method to calculate the thermal conductivity of two nanofibrous samples overlapping vertically (point crosslinking),

comparing with two nanofibrous samples placed side-by-side (facial crosslinking). We used two 14088 atoms nanofibrous systems to show the changes in thermal conductivity of different crosslinking patterns (Supplementary Fig. 38). As shown in Supplementary Fig. 39, the sample of two nanofibers overlapping vertically shows a thermal conductivity of 0.22 W m^{-1} K^{-1}, which is much lower than 0.42 W m^{-1} K^{-1} of fibers placed side-by-side, showing that the thermal conductivity of ceramic nanofibrous aerogel would be reduced significantly by changing the facial crosslinking to point crosslinking.

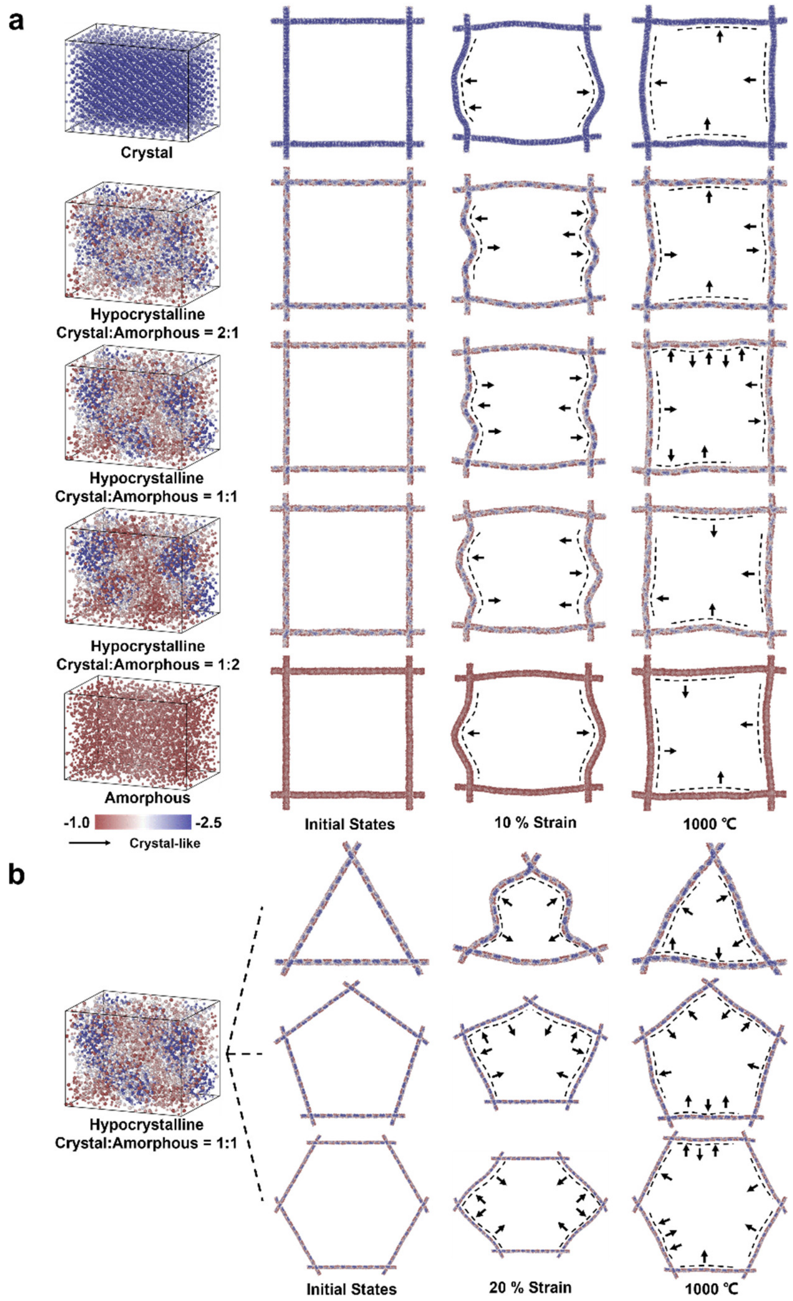

Supplementary Fig. 1 | MD simulations of crystal/amorphous/hypocrystalline nanofibers. a, Snapshots of the structural deformation under mechanical and thermal excitation in square cells, which consisted of crystal, amorphous, and hypocrystalline ceramics with different crystal/amorphous volume ratio. The colored scaled bar indicates the variation of crystalline structures from amorphous to crystal by using local entropy based fingerprint to characterize the crystallinity of each atom in the simulated system. **b**, Snapshots of the structural deformation under mechanical and thermal excitation in triangle, pentagon, and hexagon cells, which consisted of hypocrystalline ceramics with equal proportion of crystal and amorphous part.

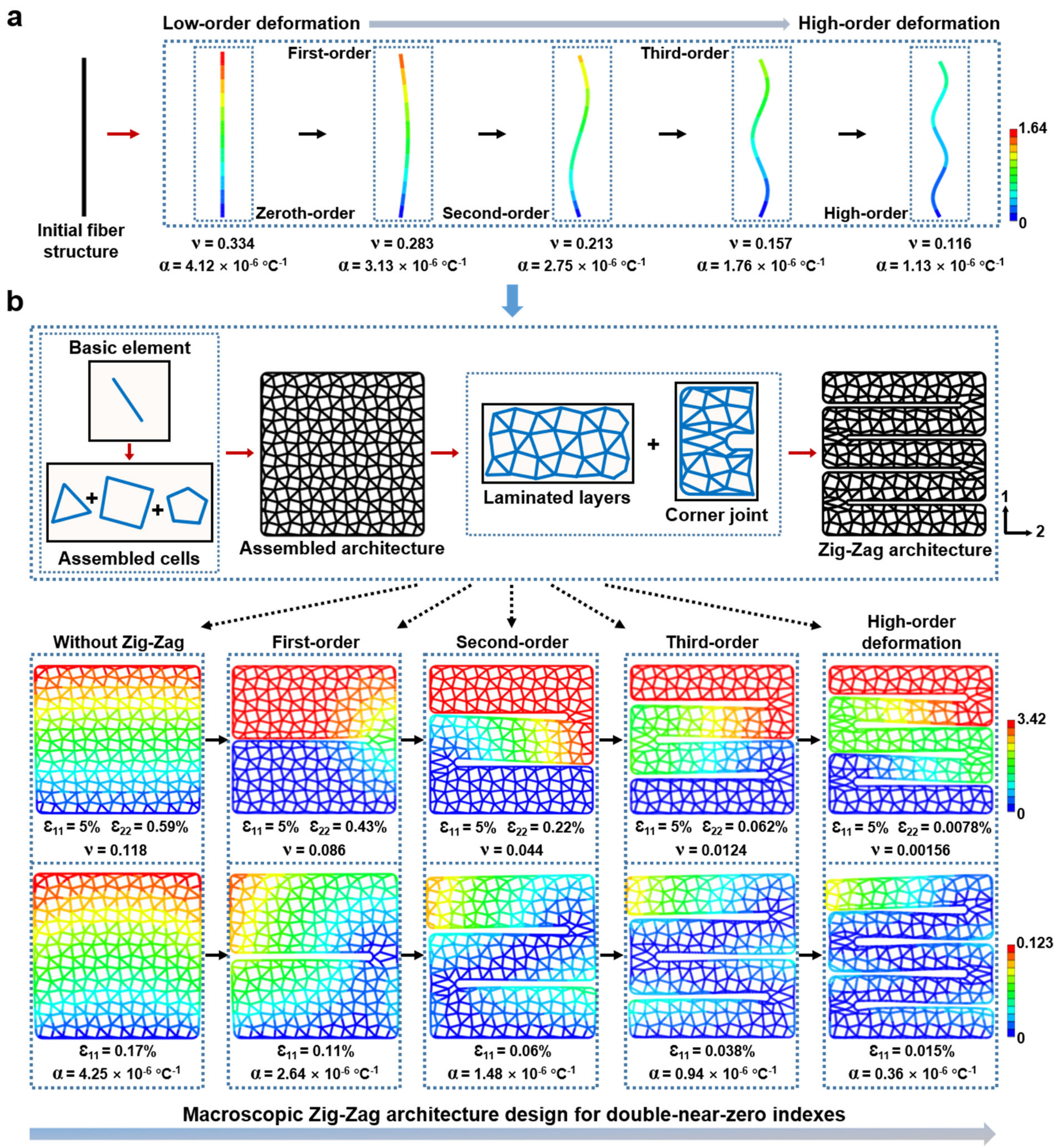

Supplementary Fig. 2 | Macroscopic architecture design of ceramic nanofibrous aerogels by FE method. a, High-order deformation mode of the beam element in accordance to the fibers in MD simulations. **b**, Analysis of the designed Zig-Zag architecture to mitigate macrostructural-induced increase of ν and α. The units of colored scale bars are millimeters, indicating absolute displacement values in ν and α calculation.

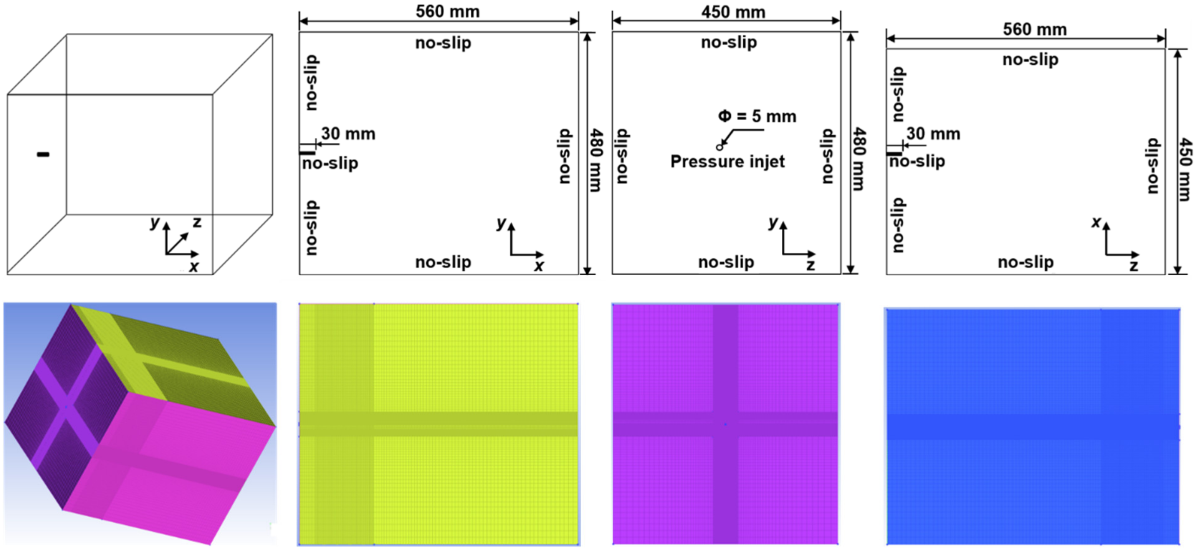

Supplementary Fig. 3 | Air turbulent flow field computational domain, boundary conditions and mesh strategies.

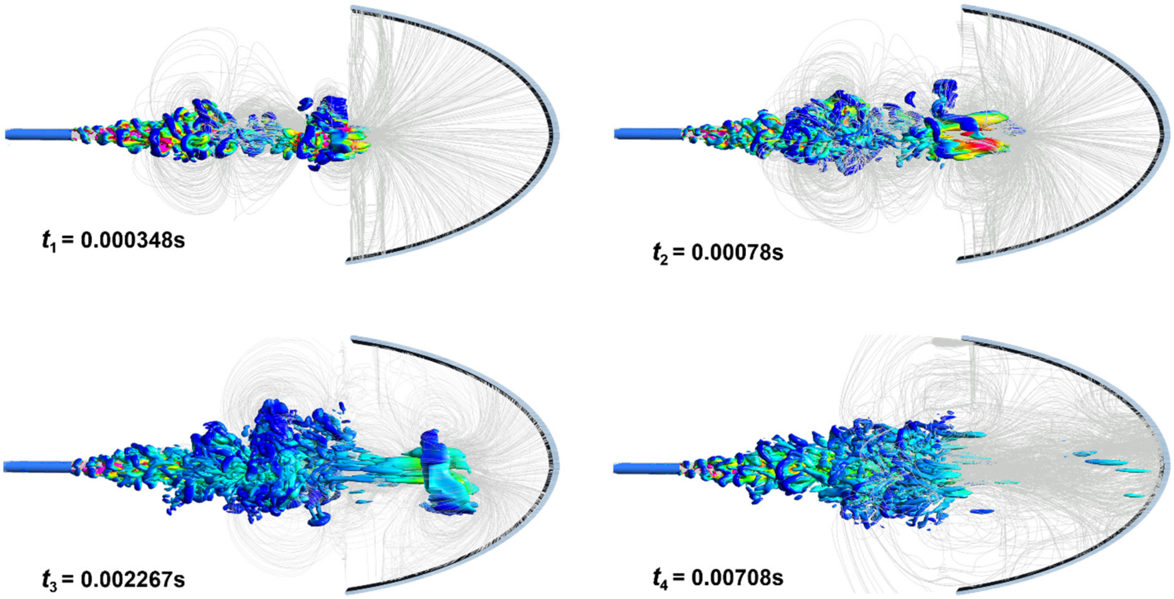

Supplementary Fig. 4 | Instantaneous turbulent structures and fibrous streamlines of the 3D turbulent flow.

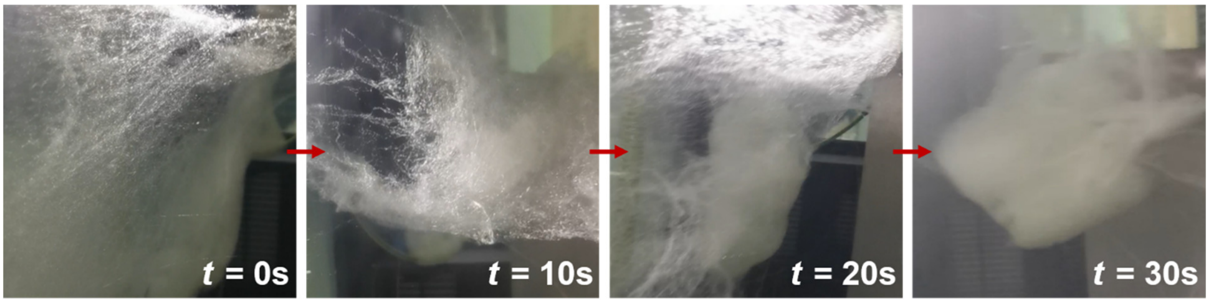

Supplementary Fig. 5 | Large-scale 3D formation process of the as-spun ceramic nanofibers by TFAE method.

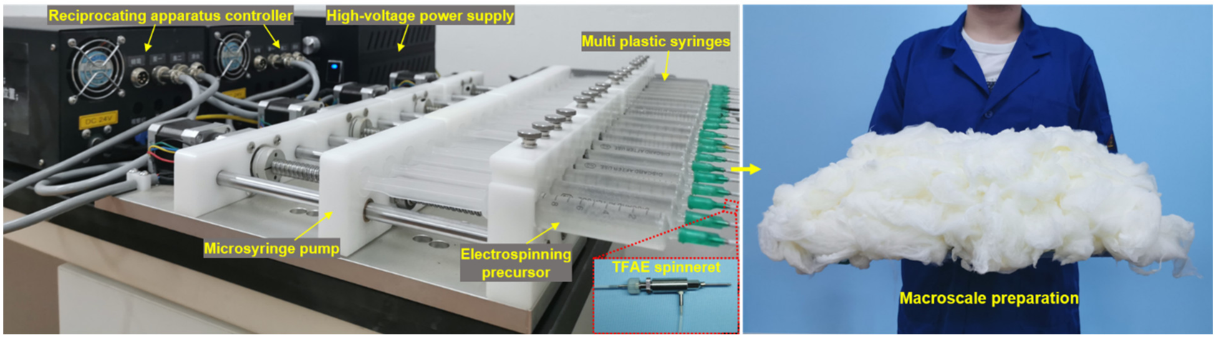

Supplementary Fig. 6 | Optical image of the TFAE fabrication system for large-scale and low-cost preparation of ZAGs. The spinning system includes microsyringe pump, air compressor (not shown in this image), TFAE coaxial spinneret (inner diameter ~0.10-0.30 mm and outer diameter ~1.0-2.0 mm), aluminum collector (not shown in this image), reciprocating apparatus controller, multi plastic syringes and high-voltage power supply.

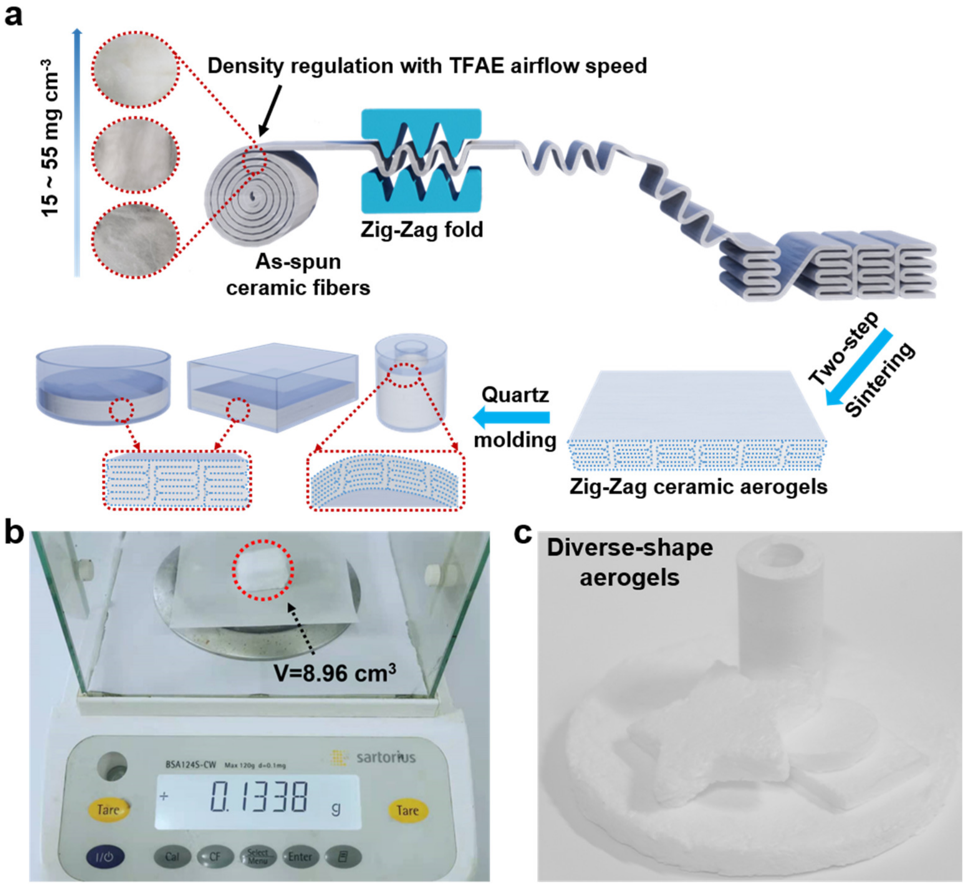

Supplementary Fig. 7 | Mechanical folding and sintering molding process of the Zig-Zag units for diverse-shape ZAGs. a, Illustration of mechanical folding and assembling process into different shapes with aerogel density regulation (15-55 mg cm^{-3}). By regulating the air flow speed from 10 to 30 m s^{-1}, the density of the as-fabricated aerogels can be easily controlled from 15 to 55 mg cm^{-3} due to the different entanglement extents. Further mechanical folding and Zig-Zig assembling will not affect the density. **b**, Density measurement for a lightweight ZAG (14.93 mg cm^{-3}). **c**, Optical image of ZAGs with diverse shapes. Compared with freeze-drying and chemical vapor deposition method, our TFAE system, by simply adding numbers of parallel coaxial electrospinning spinnerets (Supplementary Fig. 6), simultaneously combined with the automatic mechanical folding process and the two-step sintering procedure, we can efficiently achieve a low-cost and large-scale fabrication in ceramic nanofibrous aerogels with diverse shapes.

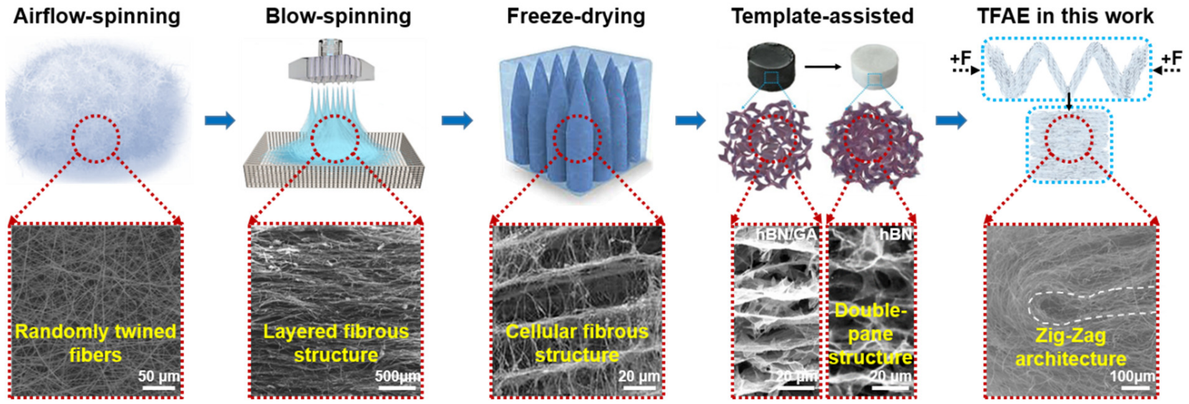

Supplementary Fig. 8 | Ceramic aerogels with different fabrications and the structural evolution. From left to right, yttria-stabilized ZrO_2 aerogel assembled with randomly twined fibers was fabricated by airflow-spinning[17]; SiO_2-Al_2O_3 aerogel assembled with the layered fibrous structure was fabricated by blow-spinning[18]; SiO_2 aerogel assembled with the cellular fibrous structure was fabricated by freeze-drying[19]; hBN aerogel assembled with the double-pane structure was fabricated by template-assisted method[20]; ZAGs in this work.

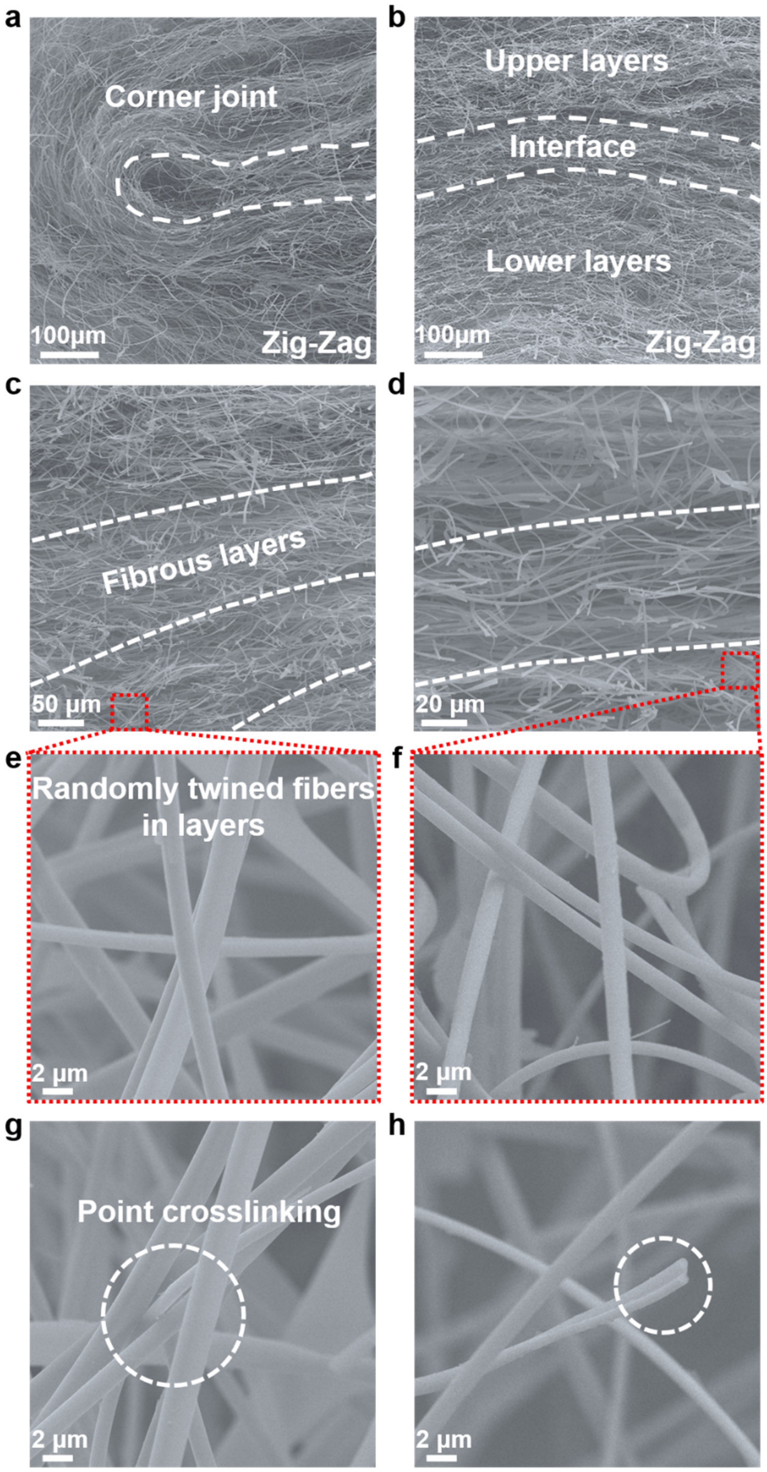

Supplementary Fig. 9 | SEM images of ZAGs. a-b, SEM images of the Zig-Zag corner joint and hierarchical layers. **c-d**, SEM images of the laminated fibrous layers. **e-f**, SEM images of the randomly twined nanofibers. **g-h**, SEM images of the point crosslinking area between ceramic nanofibers.

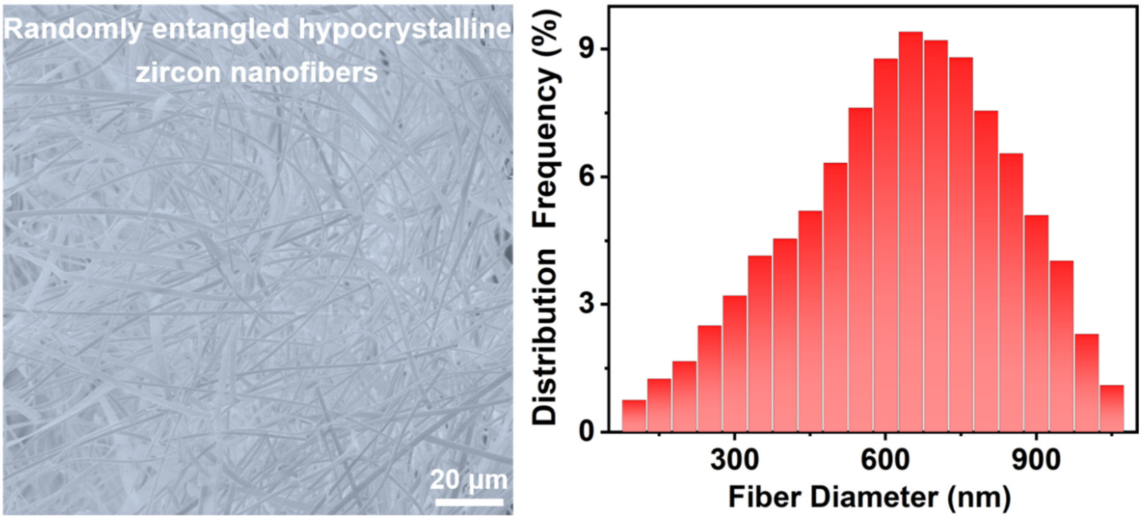

Supplementary Fig. 10 | SEM image of ZAGs and the fiber diameter distribution. According to the electrospinning and air blow spinning method for preparation of ceramic fibers[21], we can easily obtain the ceramic fibers with diameters range from hundreds of nanometers to several microns after high-temperature sintering. From our previous research, the fiber diameter from hundreds of nanometers to several microns doesn't affect the mechanical property and thermal insulation performance[21].However, if the fiber diameter is below 200 nm, which could easily lead to crystallization-induced pulverization at high temperatures with fiber fracture, resulting in poor thermal stability under extreme conditions. When the diameter increases from about 500 nm to about 2 μm, the fibers can sustain more crystallization, avoiding pulverization induced fracture. Therefore, the optimal fiber diameter in our work is from 500 nm to 1 μm for excellent thermomechanical property and high-temperature thermal insulation performance.

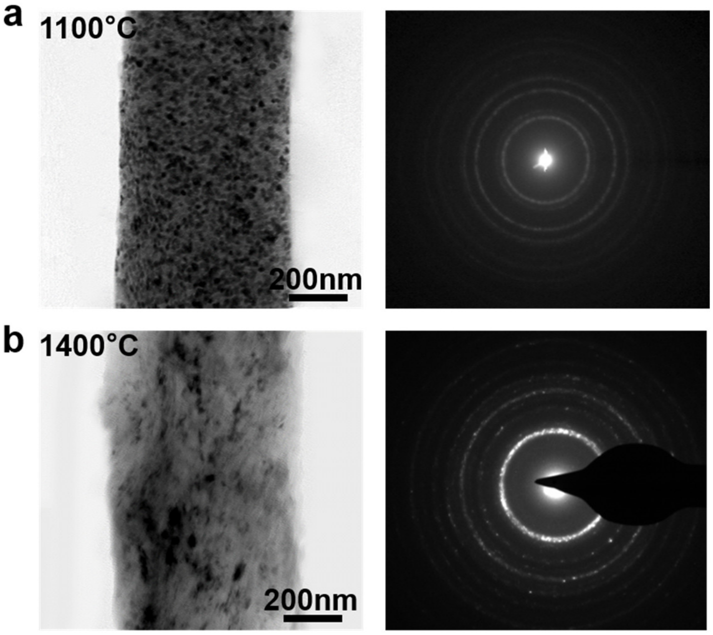

Supplementary Fig. 11 | TEM images of ZAGs. a, The morphology of a single ceramic nanofiber after thermal treatment at 1100 ℃ and the corresponding SAED. **b**, The morphology of a single ceramic nanofiber after thermal treatment at 1400 ℃ and the corresponding SAED.

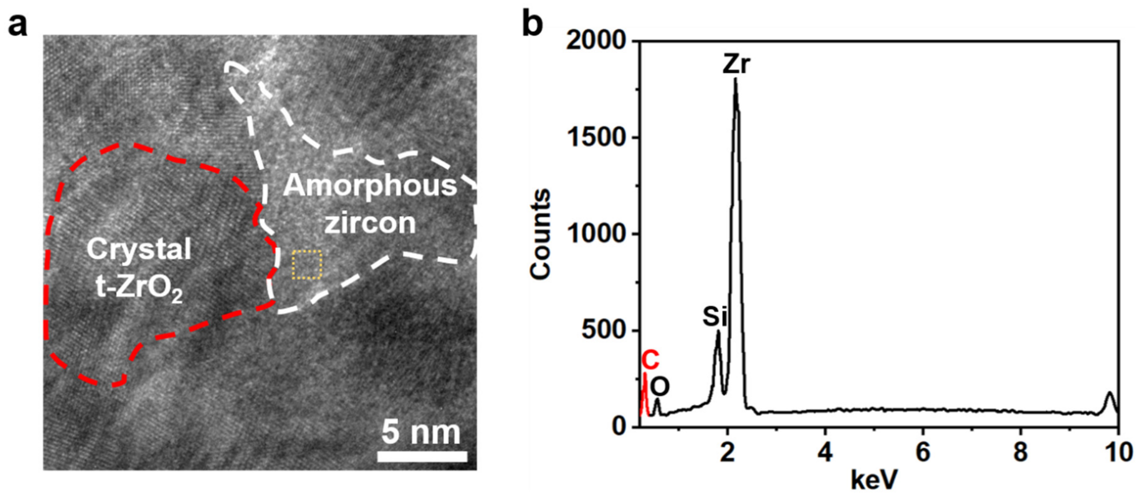

Supplementary Fig. 12 | C wrapped in the hypocrystalline Zircon. **a**, High-resolution-TEM image of the hypocrystalline zircon after thermal treatment at 1100 °C. **b**, Energy dispersive spectrum of the area marked by yellow dotted box in amorphous phase in (a). The amorphous zircon was pinned by the tetragonal zirconia with average size of ~10 nm as revealed by the high-resolution-TEM image in Fig. 2f. According to EDS of area outlined by yellow dotted box in the amorphous phase, the main compositions of amorphous phase were C, O, Si, and Zr, corresponding to the amorphous C, and zircon. Due to the good protection of hypocrystalline zircon, the embedded amorphous carbon can keep stable even at high temperatures above 1000 °C in air. The incorporation of carbon in ceramic matrix endows efficient infrared absorption under high temperatures, resulting a low thermal conductivity of ZAGs under extreme conditions.

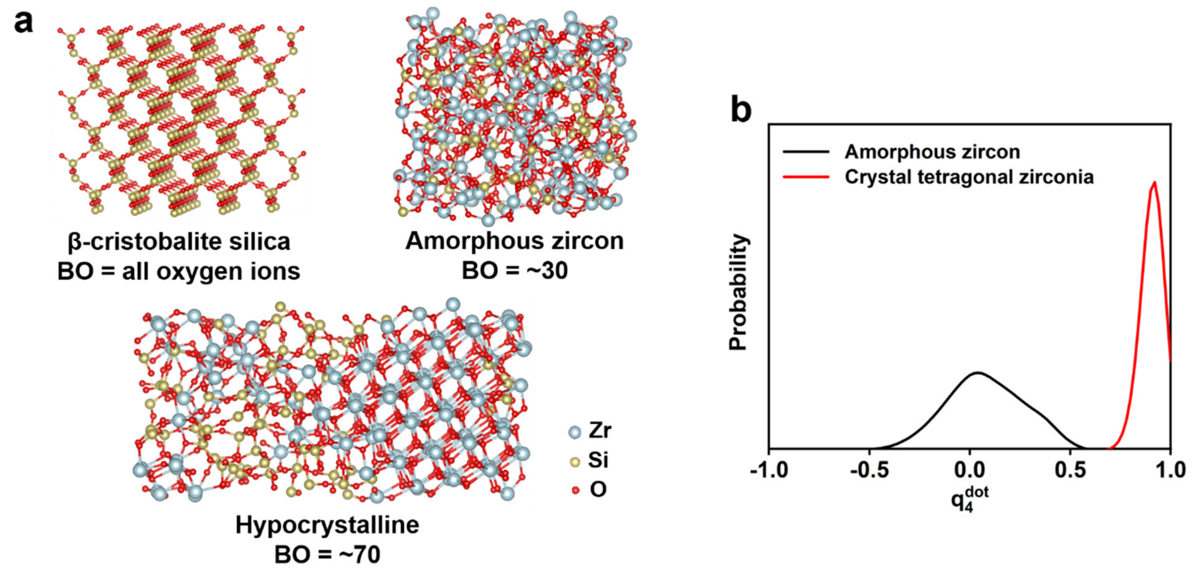

Supplementary Fig. 13 | Distinguishment of the amorphous and hypocrystalline zircon based on collective variables (CVs) we chose. a, Comparison of the number of bridging oxygen (BO) in silica, amorphous zircon, and the hypocrystalline zircon obtained by WTMetaD. **b**, The distribution of dot product of local Steinhardt's order parameter q_4^{dot} calculated at 1500 °C for amorphous zircon and tetragonal zirconia. The CVs we chose are able to distinguish amorphous and hypocrystalline zircon.

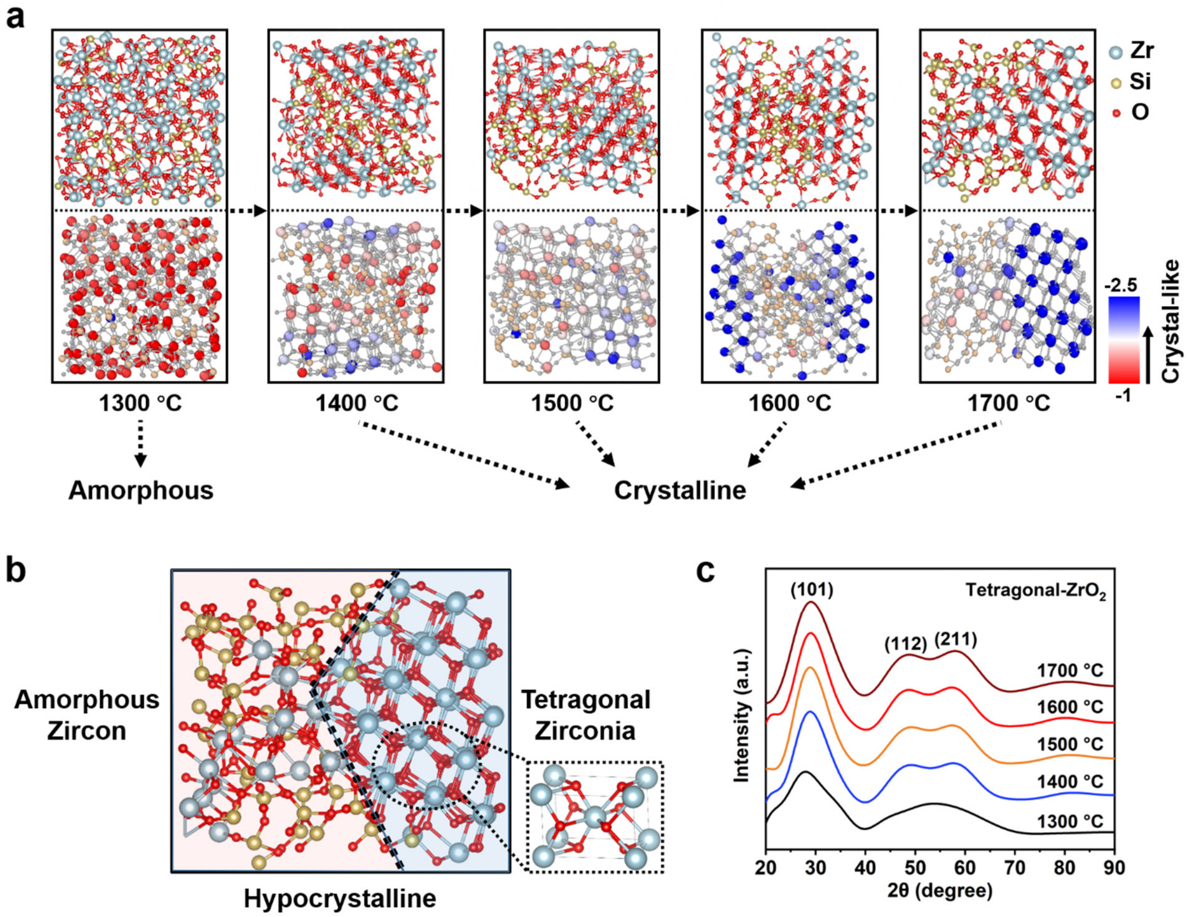

Supplementary Fig. 14 | Metadynamics for crystallization simulation. a, Snapshots of the structures obtained by WTMetaD simulation from 1300 °C to 1700 °C. The colored scaled bar indicates the variation of crystalline structures from amorphous to crystal by using local entropy based fingerprint to characterize the crystallinity of each atom in the simulated system. No crystallization process occurred at the temperature of 1300 °C, while at the temperature above 1400 °C, the system would turn into a hypocrystalline system with amorphous zircon and tetragonal zirconia. Crystallinity of the hypocrystalline zircon obtained by WTMetaD would increase with the simulation temperature. **b**, A larger version of the hypocrystalline zircon obtained at 1700 °C, with amorphous zircon at the left side and tetragonal zirconia at the opposite side. Bottom inset was the snapshot of a crystal tetragonal zirconia unit cell. **c**, The simulated XRD pattern for the samples obtained from 1300 °C to 1700 °C.

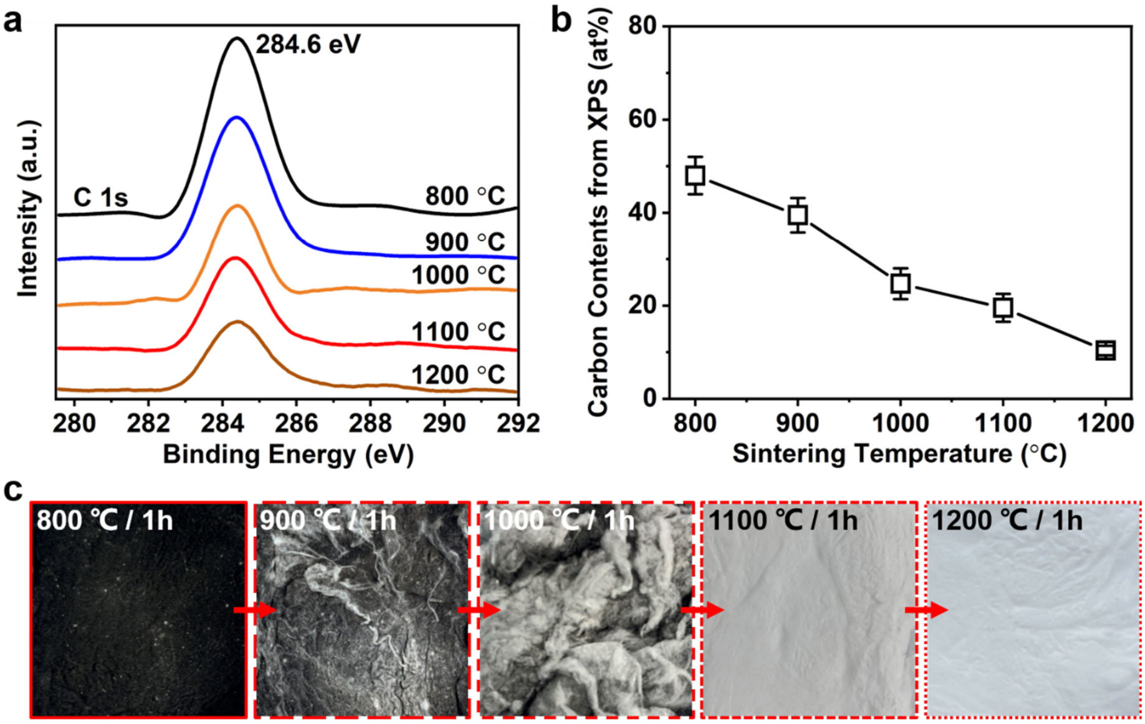

Supplementary Fig. 15 | Characterizations of ZAGs with different C contents. a, The C1s XPS characterization of ZAGs under different sintering temperatures (800-1200 ℃). **b**, C contents of ZAGs under different sintering temperatures (800-1200 ℃). Error bars indicate standard deviations of C contents. **c**, Optical images of the sample color.

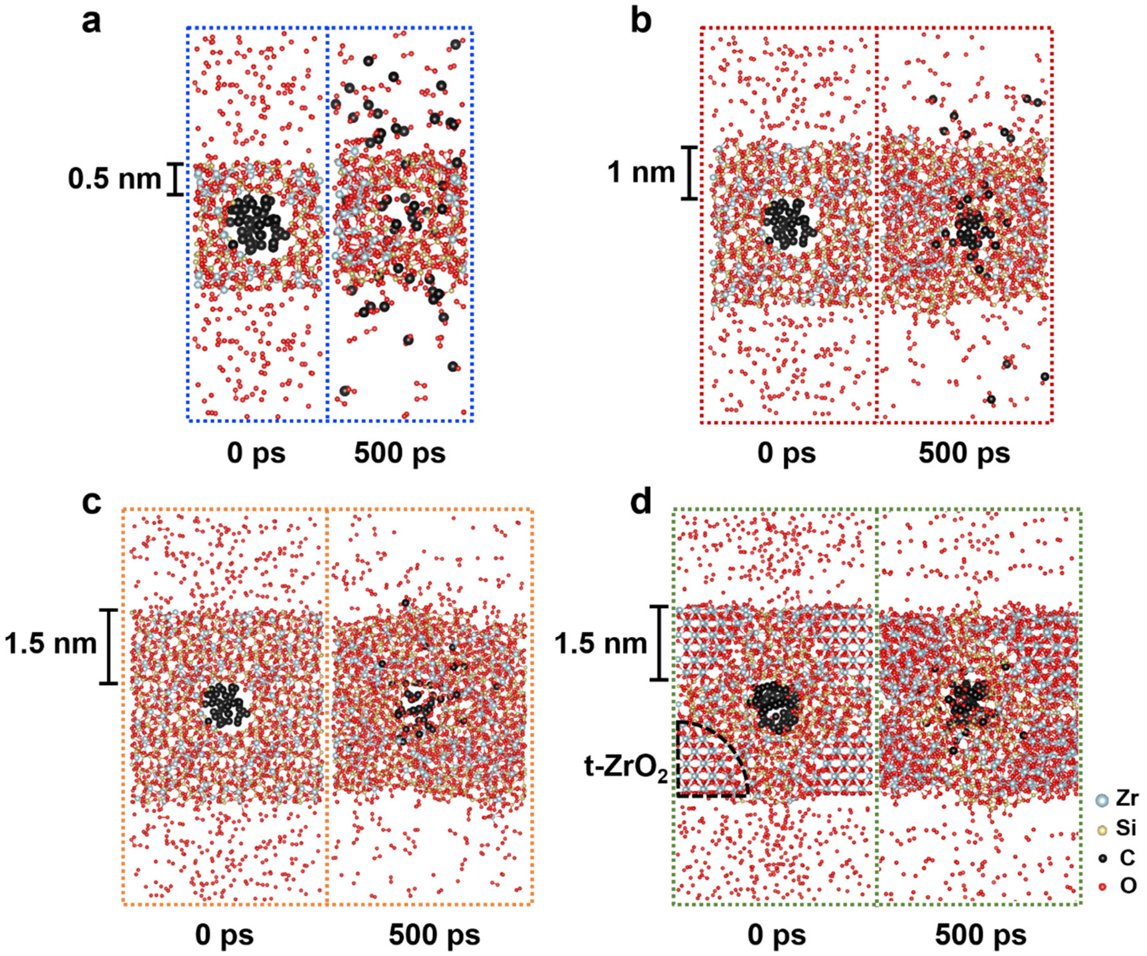

Supplementary Fig. 16 | Reactive simulation of amorphous carbon embedded in hypocrystalline zircon. Snapshots of the initial and final structures of carbon sheltered by amorphous zircon layer with the thickness of **a**, 0.5 nm, **b**,1 nm, and **c**, 1.5 nm in oxygen atmosphere. **d**, Snapshots of the initial and final structures of hypocrystalline zircon to protect the internal carbon atoms. All four systems were sintered at the temperature of 1100 °C for 500 ps.

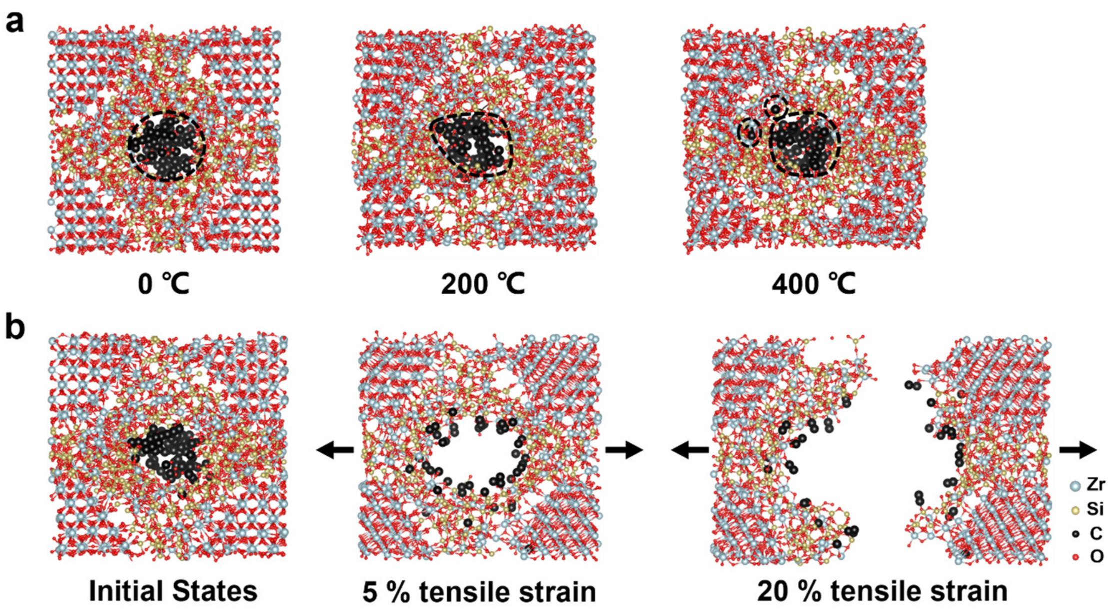

Supplementary Fig. 17 | Thermal and mechanical properties affected by interface between amorphous carbon and hypocrystalline zircon. a, under thermal treatment and **b**, tension. We further examined the interface between amorphous carbon and hypocrystalline zircon on the thermal and mechanical properties of fibrous aerogel. To better show the contact between carbon atoms with amorphous zircon, we chose the hypocrystalline sample as discussed in the section e) of MD simulations of hypocrystalline zircon, which has higher carbon proportion than that in experiment. Thermal expansion and fracture simulations were performed with the same configuration as that in section c) and d). As shown in Supplementary Fig. 17, the region formed by carbon atoms deforms, and the interface between carbon and hypocrystalline zircon blends together under thermal treatment. Some carbon atoms may migrate into amorphous zircon at the temperature of 400 °C. When under tensile stress, the fracture tends to occur in the carbon region, and crack then grows along the interface between amorphous zircon and tetragonal zirconia. In general, this interface doesn't affect aerogel thermal stability but may reduce the tensile property.

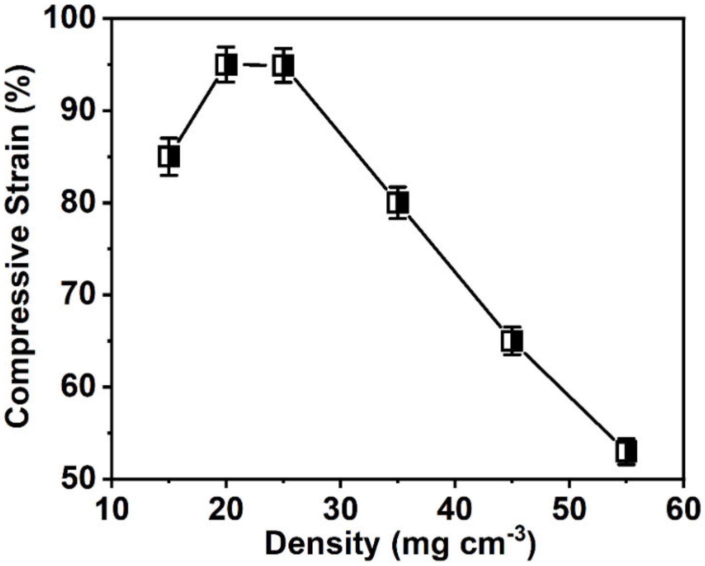

Supplementary Fig. 18 | Compression resilience of ZAGs as a function of density. Standard deviations of compression properties are shown as error bars.

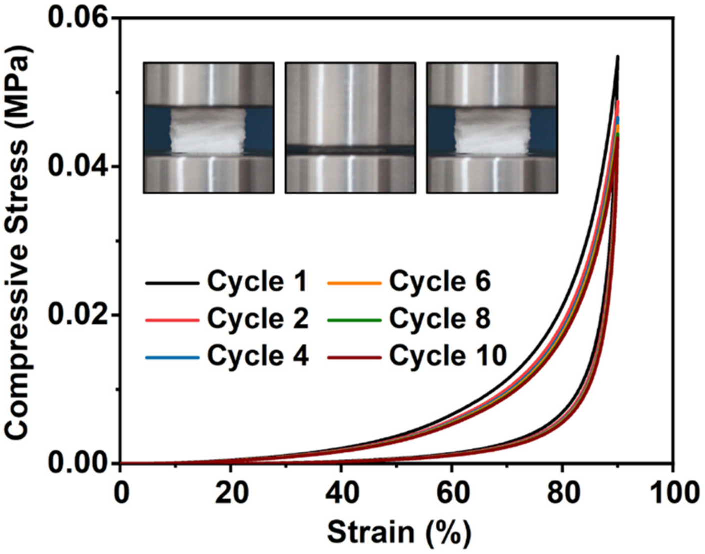

Supplementary Fig. 19 | Stress-strain curves of ZAGs at 90% strain for 10 cycles.

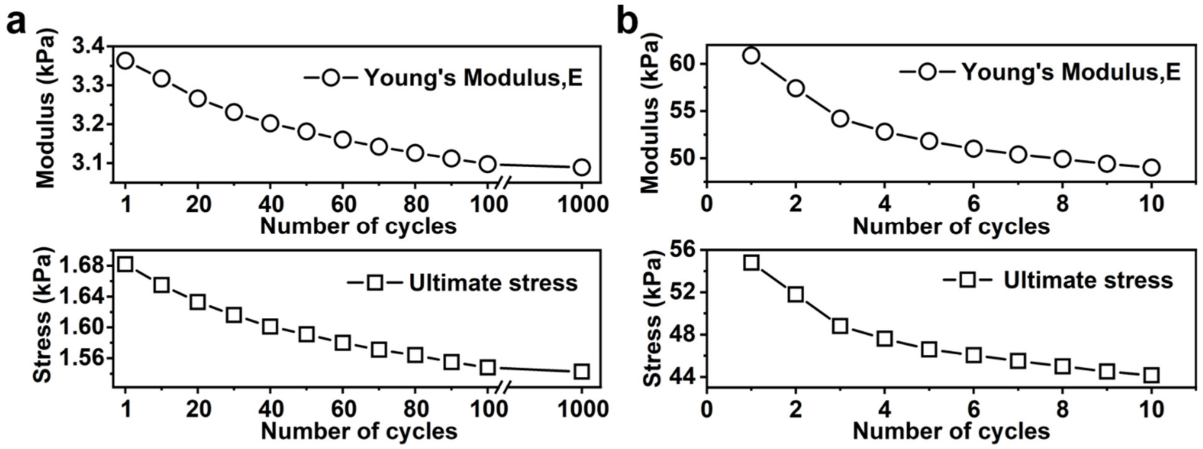

Supplementary Fig. 20 | Compressive properties of ZAGs. a, The Young's modulus and ultimate stress at 50% strain for 1000 compression cycles. **b**, The Young's modulus and ultimate stress at 90% strain for 10 compression cycles.

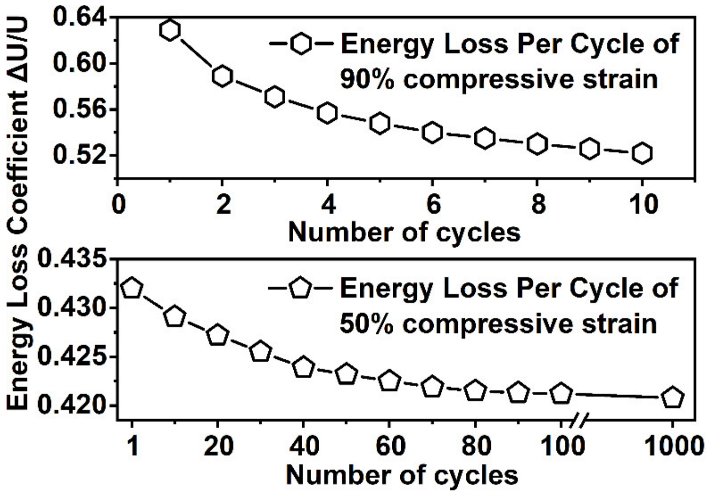

Supplementary Fig. 21 | Energy loss coefficient at 50% compressive strain for 1000 compression cycles and at 90% compressive strain for 10 cycles.

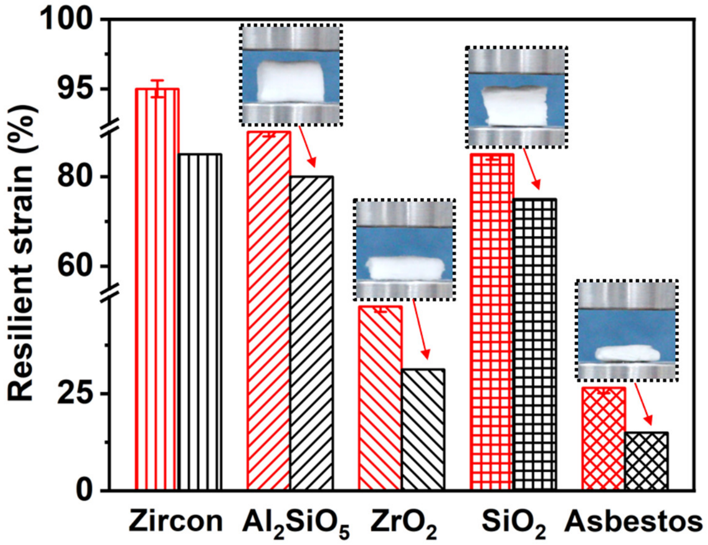

Supplementary Fig. 22 | Comparison of resilience in hypocrystalline zircon, Al_2SiO_5, ZrO_2, SiO_2 and asbestos with (red stripes) or without (black stripes) Zig-Zag architecture. Error bars indicate standard deviations of mechanical resilience in different samples with Zig-Zag architecture.

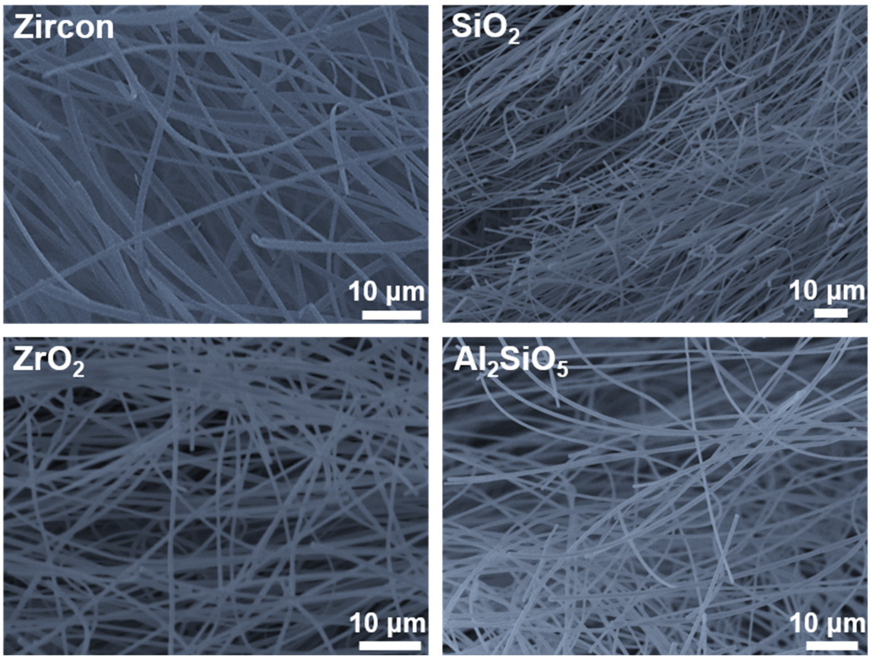

Supplementary Fig. 23 | SEM images of the entangled hypocrystalline zircon, Al_2SiO_5, ZrO_2 and SiO_2 in Zig-Zag architecture to show the universality of our TFAE fabrication method.

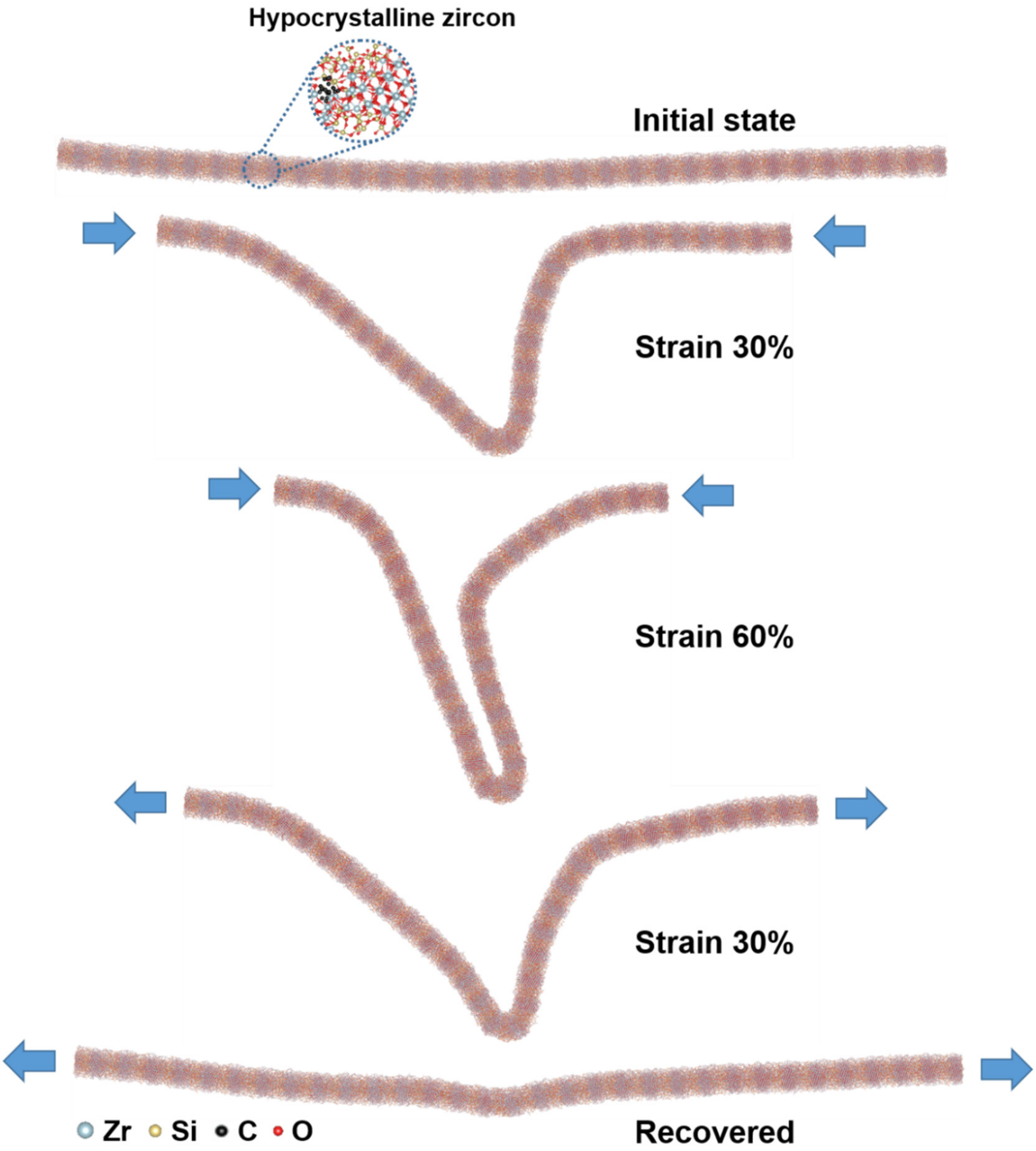

Supplementary Fig. 24 | Snapshots of the bending process by MD simulation (The bendability of single nanofiber with gradient strain).

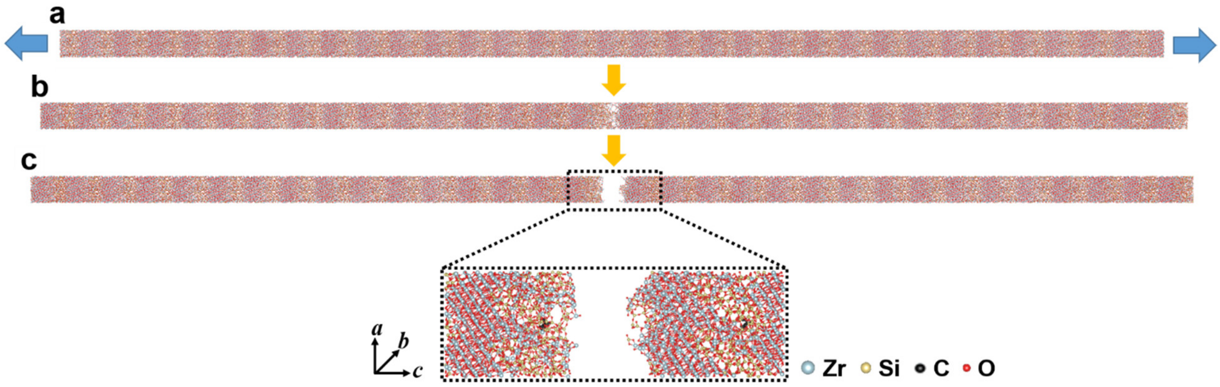

Supplementary Fig. 25 | Snapshots of the fracture process by MD simulation. a, Initial configuration. **b**, Fibrous structure at 6.6% tensile strain. **c**, Final fracture state of the global fibrous structure.

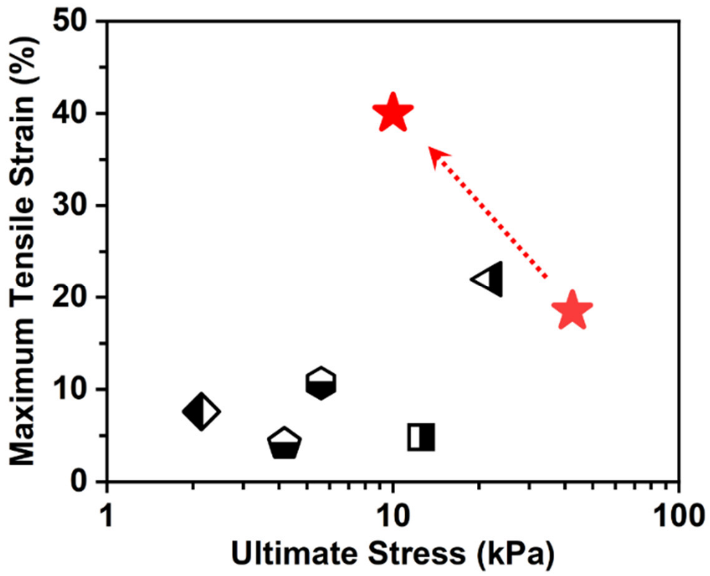

Supplementary Fig. 26 | The maximum tensile strain and ultimate stress of ZAGs compared with other ceramic nanofibrous aerogels. Red pentagram, ductile fracture with strain from 18.5% to 40% of ZAGs in this work; diamond, SiO_2 fiber combined with SiO_2 granular nanoparticles[22]; pentagon, SiO_2 fiber with binder[19]; hexagon, SiO_2 fiber with binder[23]; square, SiO_2 fiber with graphene[24]; top triangle, SiC-SiOx nanowire[25].

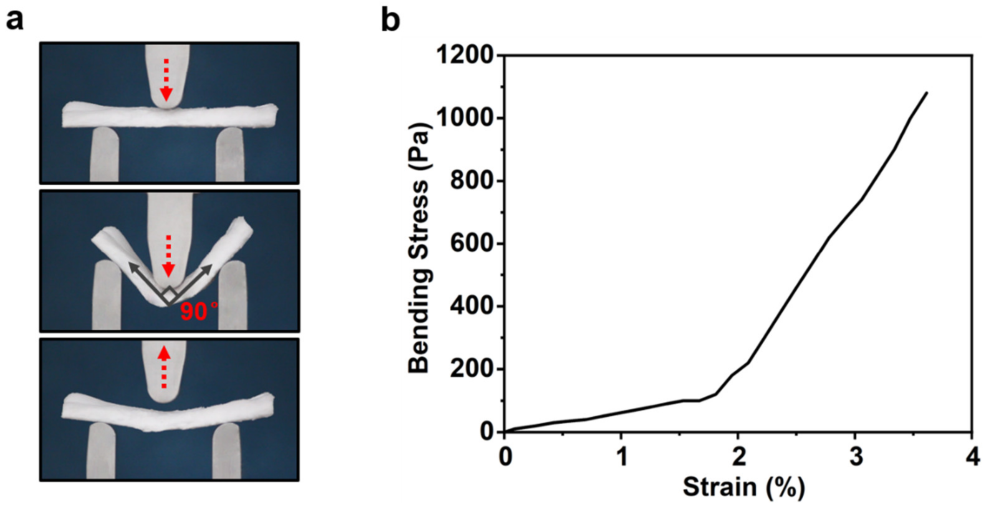

Supplementary Fig. 27 | Three-point bending test of ZAGs. a, Experimental snapshots of the bending experiment. **b**, Three-point bending stress–strain curve of ZAGs. The sample showed a large recoverable bending angle up to 90°, presenting a remarkable bendability of our aerogels.

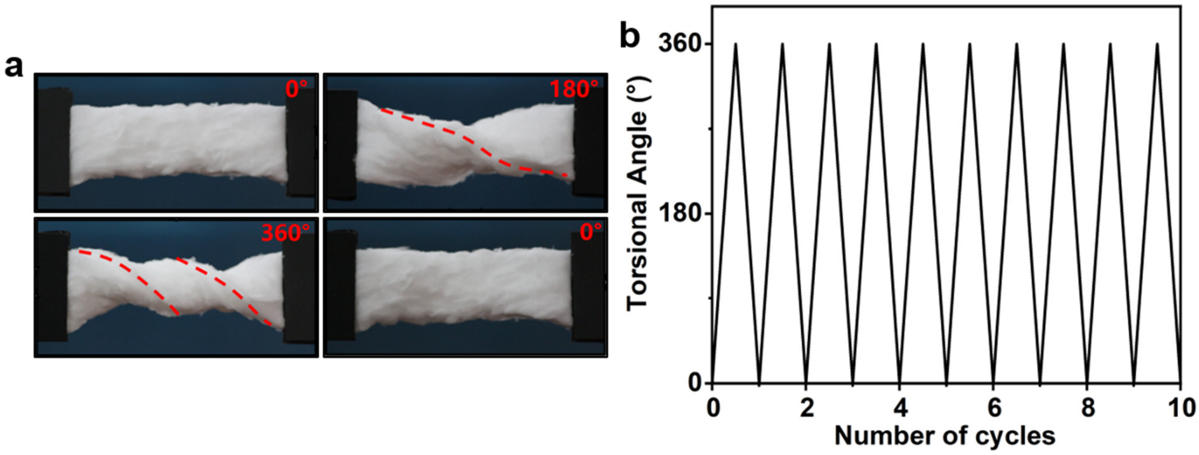

Supplementary Fig. 28 | Torsion test of ZAGs. a, Experimental snapshots of the torsion experiment. **b**, Torsional curve of torsion angle versus torsion cycles.

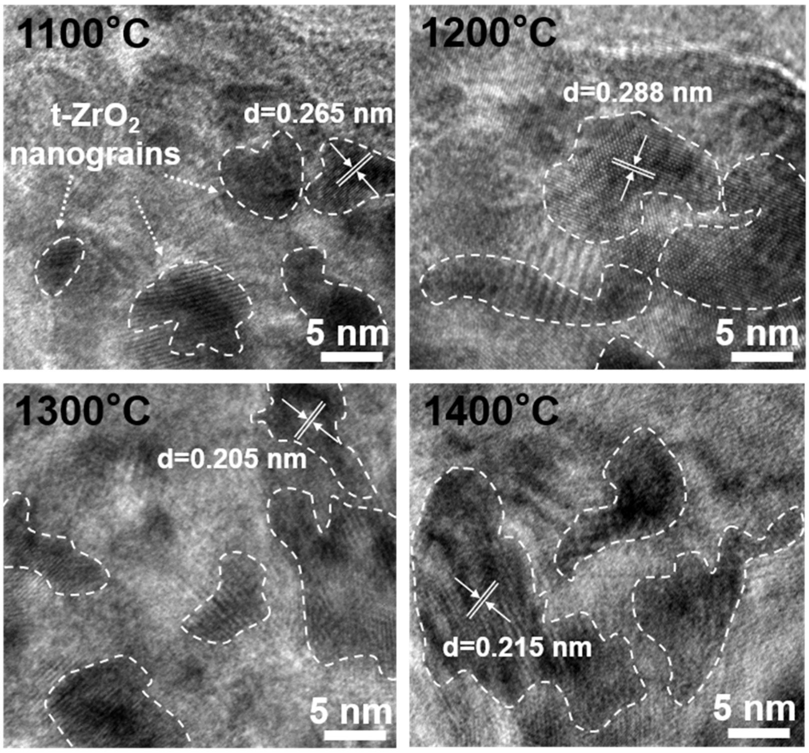

Supplementary Fig. 29 | High-resolution TEM images of ZAGs with hypocrystalline zircon after thermal treatment at 1100, 1200, 1300, and 1400 °C. The TEM characterizations show the size of the tetragonal zirconia nanograins gradually grow up with the increase of sintering temperature. When the sintering temperature is below 1300 °C, the grain size of t-ZrO_2 is less than 10 nm, indicating a good thermomechanical stability. However, when the sintering temperature reaches 1400 °C, the grainsize is increased, which slightly weakens the thermal stability of ZAGs.

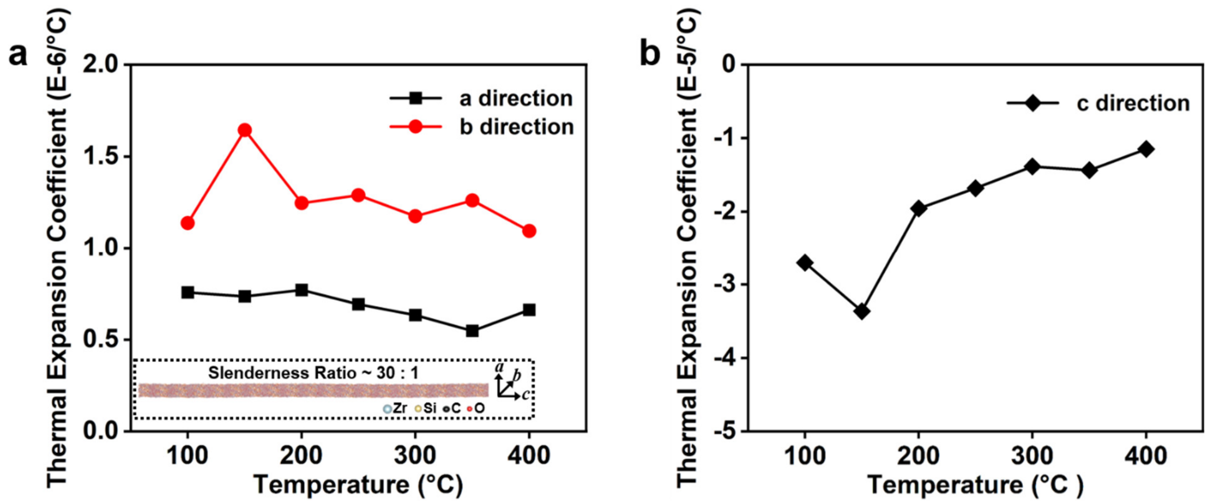

Supplementary Fig. 30 | MD simulations of thermal expansion with the increase of temperature. a, Positive α along a and b direction (radial). **b**, Negative α along c direction (axial).

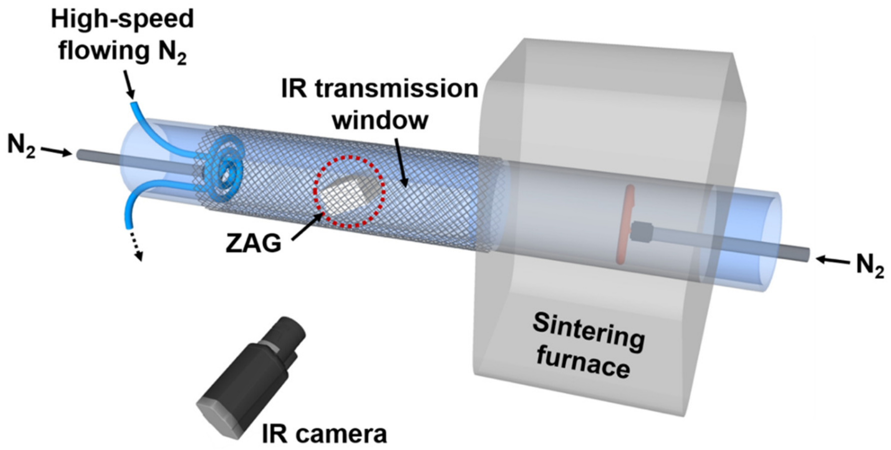

Supplementary Fig. 31 | A special designed thermal shock testing system for ceramic aerogel materials. Two pistons were fixed at both ends of the quartz tube. The left piston was cooled by high-speed flowing N_2 at the ambient temperature as cold source, and the right one was heated by furnace at 1000 °C as hot source. The ceramic aerogel was moved between the cold and hot sources by N_2 gas of the pneumatics devices. One IR transmission window was opened at the middle of the tube for IR measurement. By controlling the gas flow of the pneumatics devices, the ceramic aerogels can be heated or cooled easily with different frequencies to realize the thermal shock test for thousands of cycles. We swiftly drove the ZAGs back and forth between the hot and cold sources by switching the pneumatic devices at the tube ends, rapidly heated the sample to 1000 °C and then cooled to ambient temperature with a frequency of 0.07 Hz and temperature variation speed up to 200 °C s^{-1}.

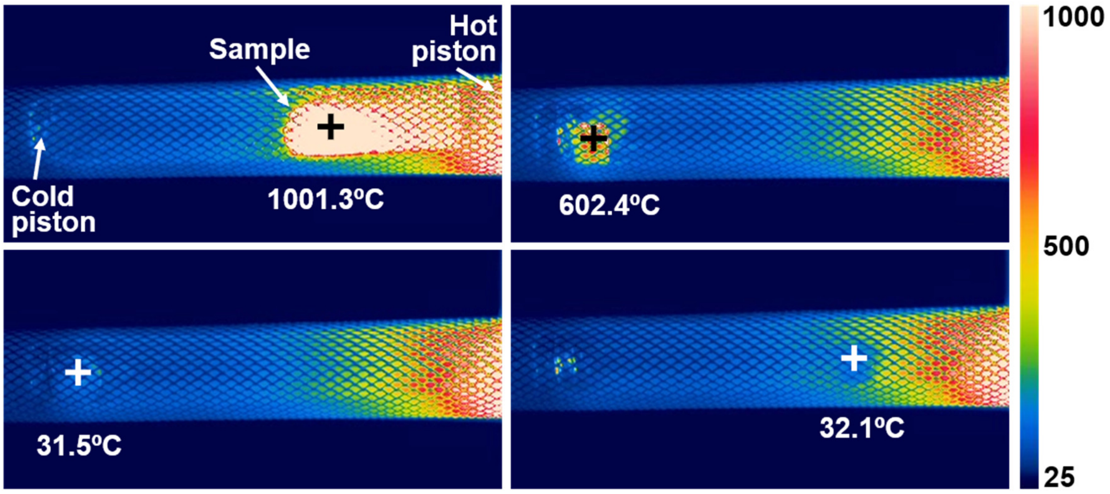

Supplementary Fig. 32 | Infrared images of ZAGs in one thermal shock cycle by using the home-made pneumatic thermal shock testing system.

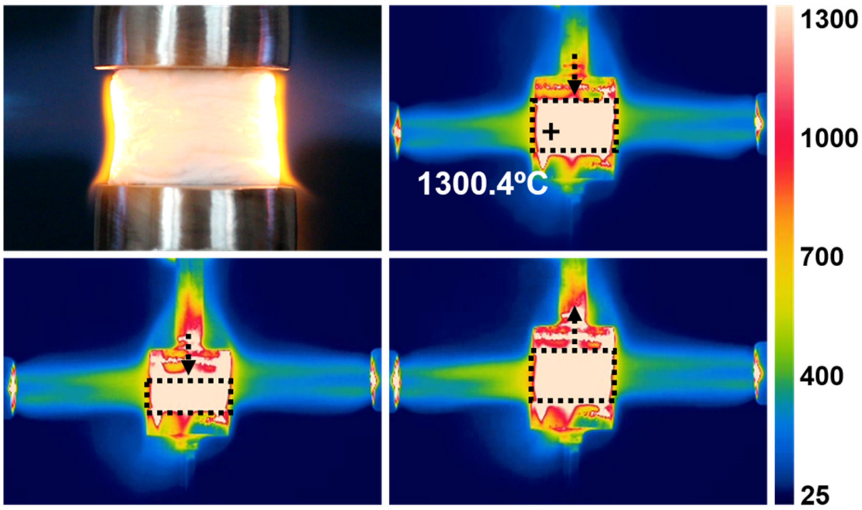

Supplementary Fig. 33 | Infrared images of ZAGs in one cycle of compression and recovery process at 50% compressive strain under the bilateral flame of butane blowtorch.

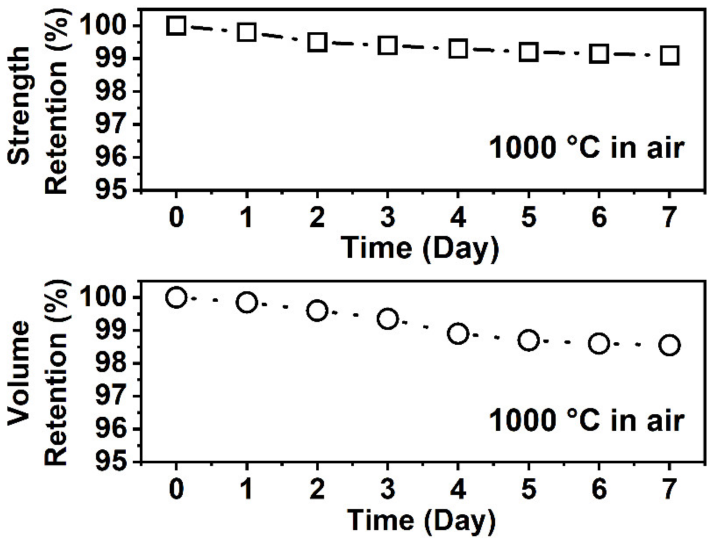

Supplementary Fig. 34 | Strength and volume variation of ZAGs in long-term high temperature exposure.

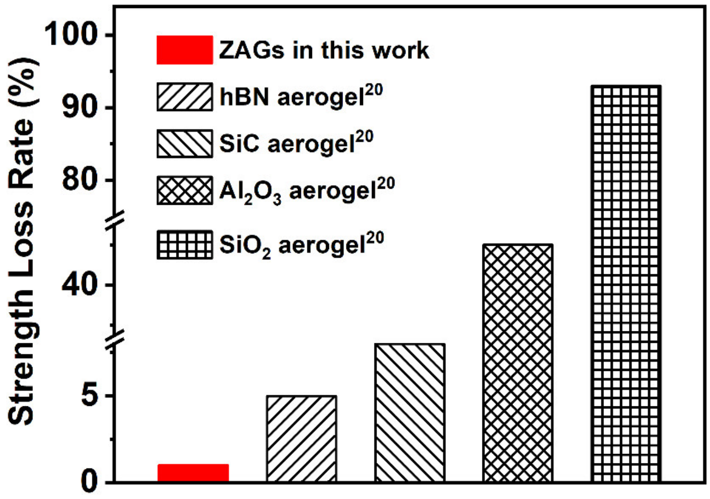

Supplementary Fig. 35 | Strength degradation of ZAGs after thermal shocks compared with other ceramic aerogels.

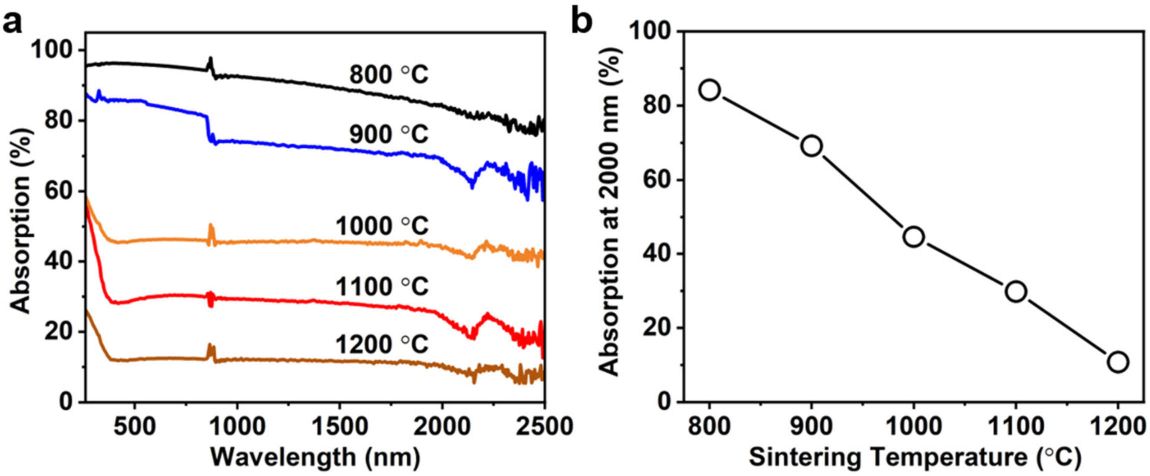

Supplementary Fig. 36 | Influence of C contents to high-temperature thermal radiation absorption. a, IR absorption spectrum of ZAGs. **b**, The IR absorption of samples with different C contents at the wavelength of 2000 nm.

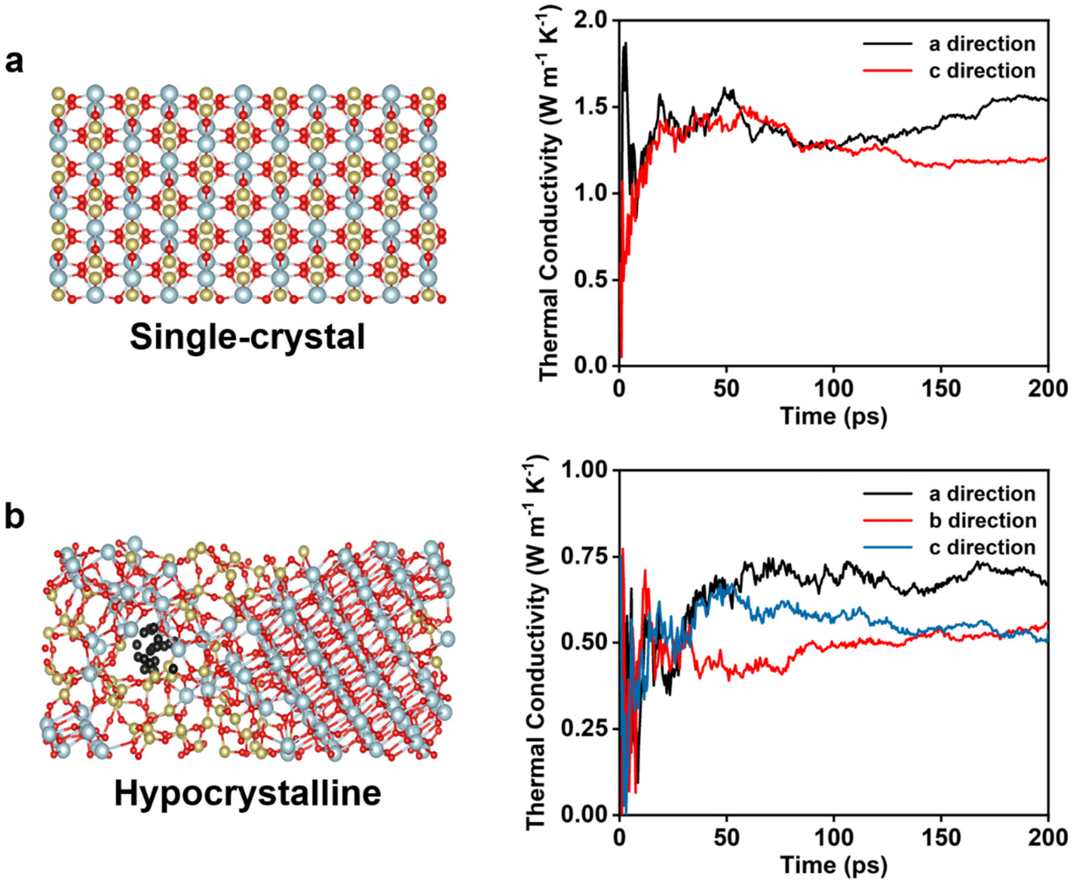

Supplementary Fig. 37 | Time dependence of thermal conductivity along different direction obtained by direct method. a, The *a* and *c* direction for the single-crystal zircon. **b**, The *a*, *b* and *c* direction for the hypocrystalline zircon.

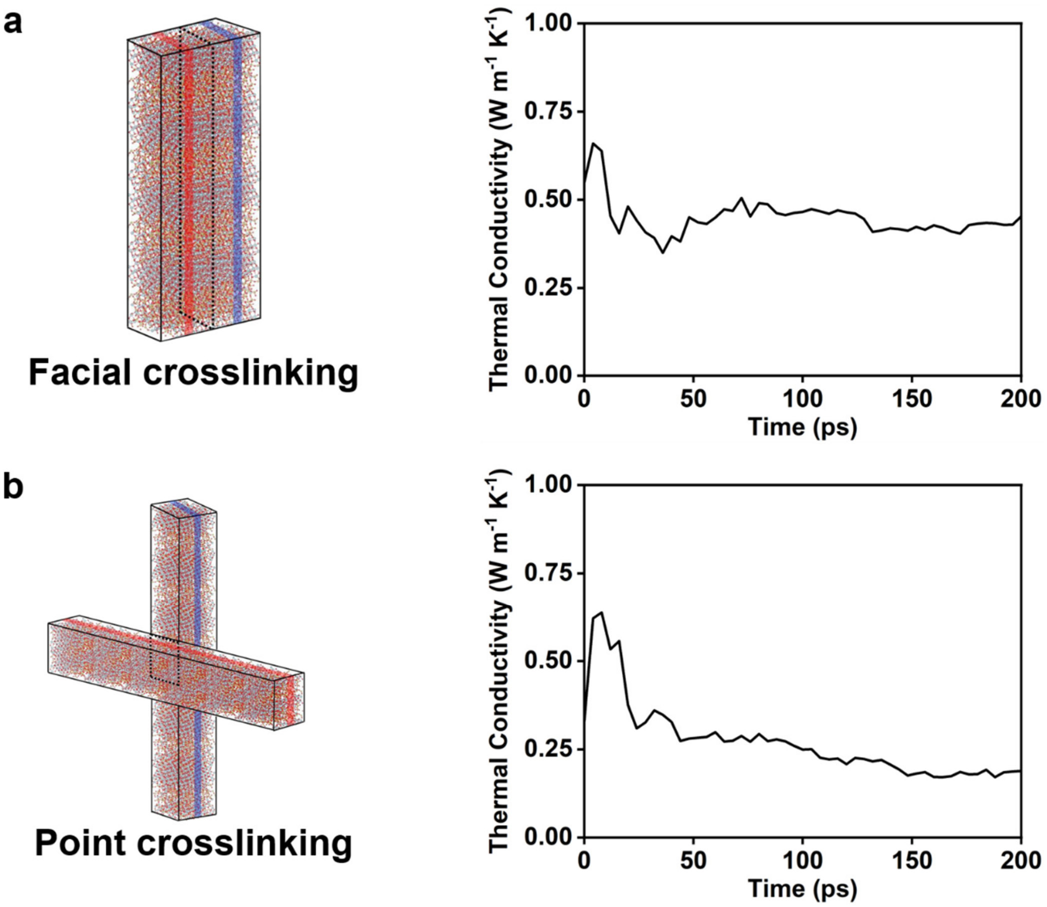

Supplementary Fig. 38 | Time dependence of thermal conductivity of two different crosslinking form of hypocrystalline nanofibers by direct method. a, Facial crosslinking pattern. **b**, Point crosslinking pattern.

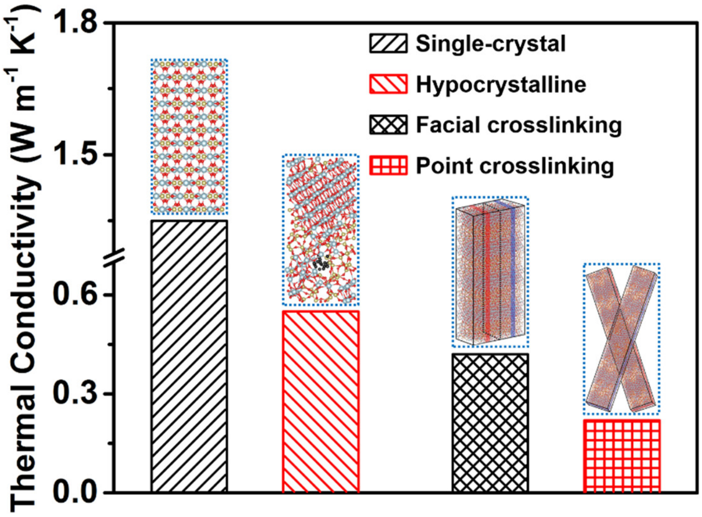

Supplementary Fig. 39 | MD simulations of the reduction of thermal conductivity with different phase structures and assemble patterns. To further understand the low κ at room temperatures, we performed MD simulations to explain the reduction of the κ in different phase structures and crosslinking patterns. According to our simulations (Supplementary Fig. 37-38), we found this low κ at room temperature can be attributed to the enhanced phonon scattering in the hypocrystalline zircon, which has efficiently decreased the κ from 1.35 W m^{-1} K^{-1} of single crystal structure to only 0.55 W m^{-1} K^{-1}. Moreover, the randomly twined fibrous structure with point crosslinking pattern suppressed the phonon transmission of solid conduction, decreasing the κ from 0.42 W m^{-1} K^{-1} of facial crosslinking pattern to only 0.22 W m^{-1} K^{-1}.

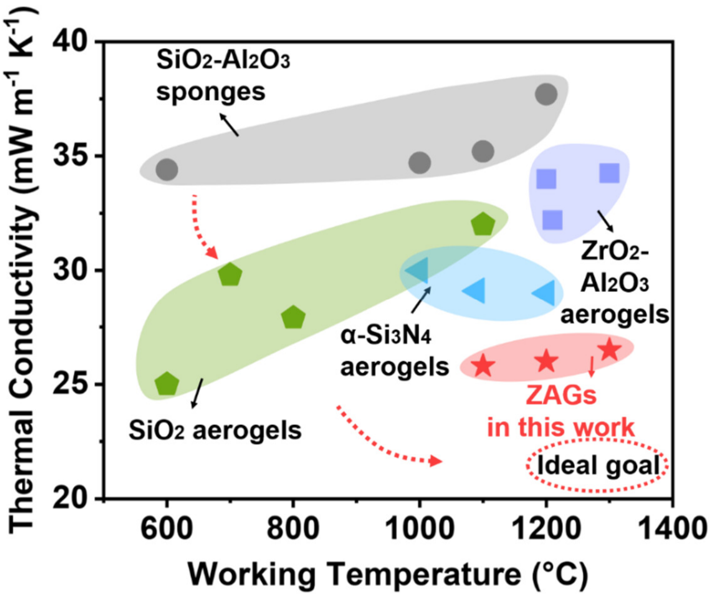

Supplementary Fig. 40 | Room temperature thermal conductivity in air versus working temperature for ceramic nanofibrous aerogels. Red pentagram, ZAGs in this work; circle, SiO_2-Al_2O_3 fibrous sponges[18]; pentagon, SiO_2 fibrous aerogels with binder[19]; square, ZrO_2-Al_2O_3 fibrous aerogels[26]; triangle, α-Si_3N_4 nanobelt aerogels[27]. Comparing with state-of-the-art ceramic fibrous aerogels, our ZAGs exhibit κ among the lowest value at room temperature with robust thermomechanical stability up to 1300 °C. We note our comparison here is limited to nanofibrous aerogel, and lower RT κ of 22 mW m^{-1} K^{-1} have been achieved in specifically designed hBN aerogel[20].

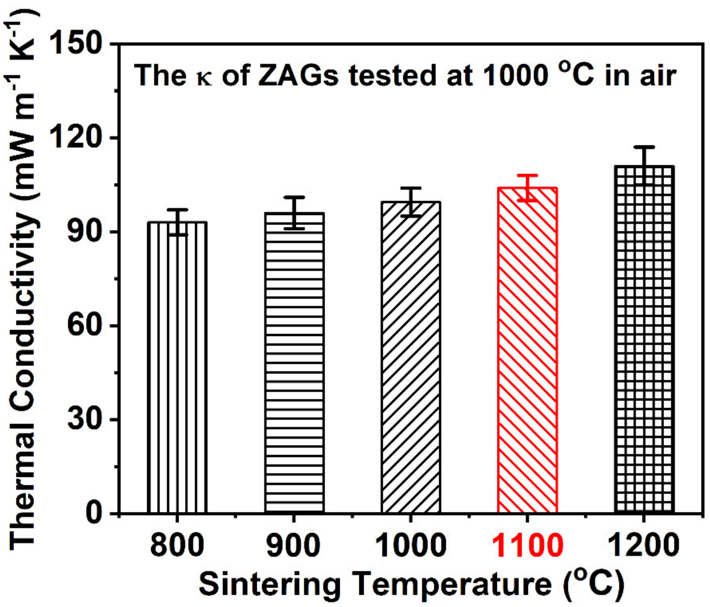

Supplementary Fig. 41 | High temperature thermal conductivities of samples with different C contents (sintering temperature from 800 °C to 1200 °C). Error bars indicate standard deviations of κ.

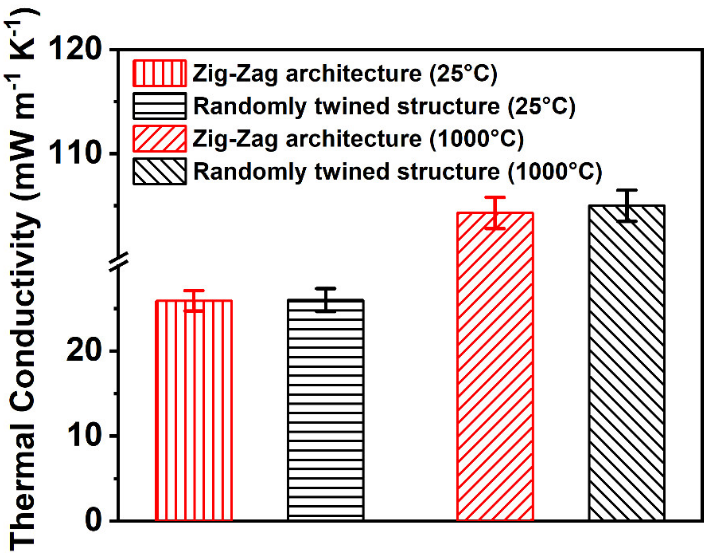

Supplementary Fig. 42 | Comparison of thermal conductivity between the Zig-Zag ZAGs and randomly twined ZAGs (25 °C and 1000 °C in air). Error bars indicate standard deviations of κ. According to our experimental test, our ZAGs exhibit a comparable κ in two different structural forms. Moreover, from our previous studies[21], ceramic aerogels can remarkably increase their thermal resistance by reducing the mean free path of phonons through enhancing phonon scattering and propagation redirection, and suppressing phonon transmission among units by introducing more van der Waals contacts and interface gaps. As a result, the solid conduction in ceramic aerogels can be effectively reduced to endow an outstanding thermal insulating performance beyond the reach of conventional insulating materials. For example, our ZAGs with a density of 15 mg cm^{-3} exhibit a low thermal conductivity (κ) of 26 mW m^{-1} K^{-1} at ambient condition, which slightly increase to only 27.3 mW m^{-1} K^{-1} with density up to 55 mg cm^{-3} (Fig. 4d, Supplementary Table 1), presenting an excellent density-insensitive property.

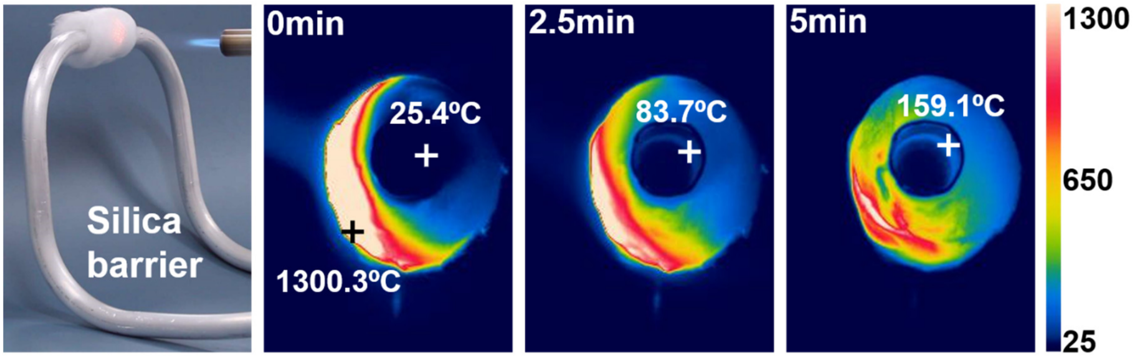

Supplementary Fig. 43 | Thermal insulation performance of conventional silica fibrous barrier for aero engine (CFM56) fuel tube under the flame of butane blowtorch.

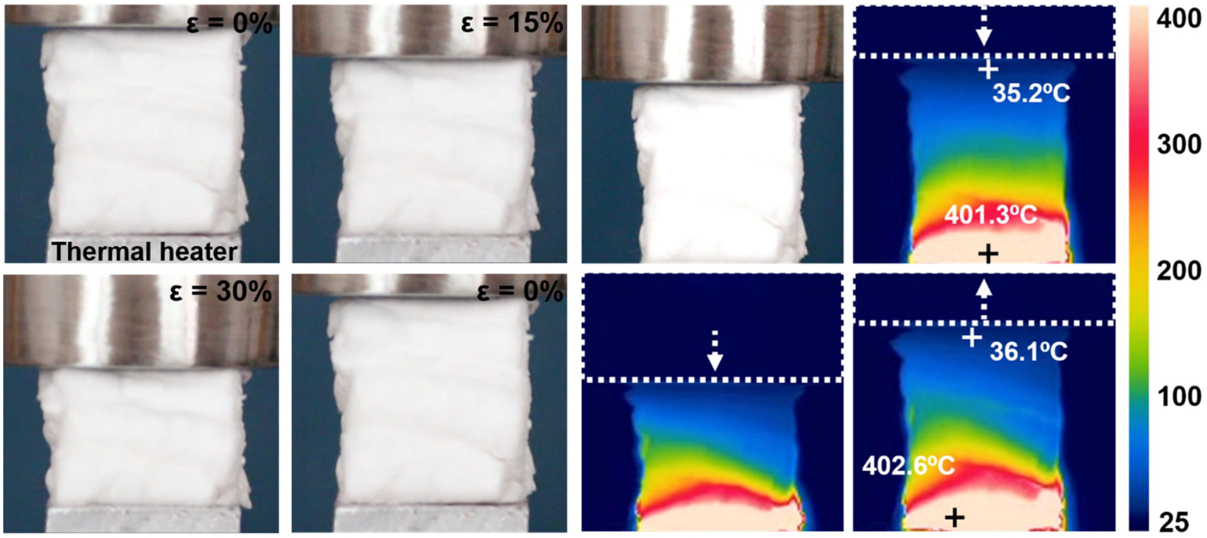

Supplementary Fig. 44 | Thermal insulation performance of ZAGs at 400 °C under vibrations.

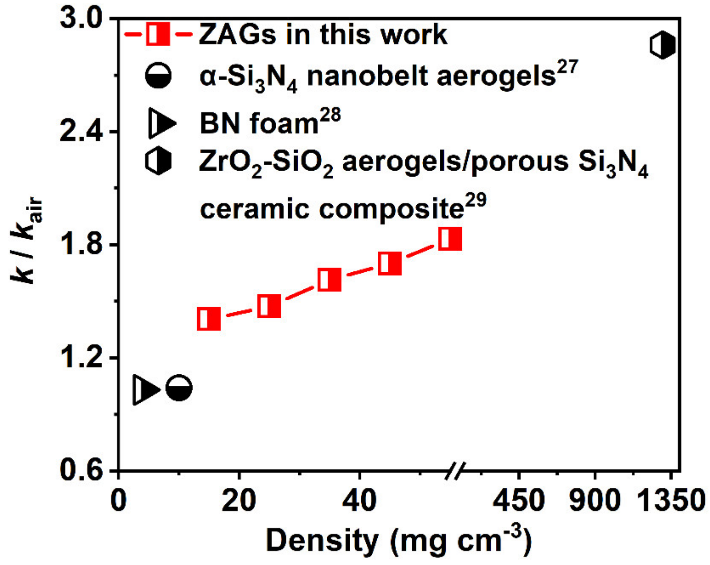

Supplementary Fig. 45 | Permittivity of the ZAGs at 100 kHz to that of air as a function of the density, and comparison with other ceramic aerogels. The high porosity and electric insulation of the Zig-Zag zircon nanofibrous building blocks, making our ZAGs a good material with low permittivity. We investigated the dielectric constant of ZAGs relative to air at 100 kHz with different densities (from 15 to 55 mg cm^{-3}). As shown in Supplementary Fig. 45, the permittivity relative to air increases slightly from 1.405 under 15 mg cm^{-3} to 1.831 at 55 mg cm^{-3}.

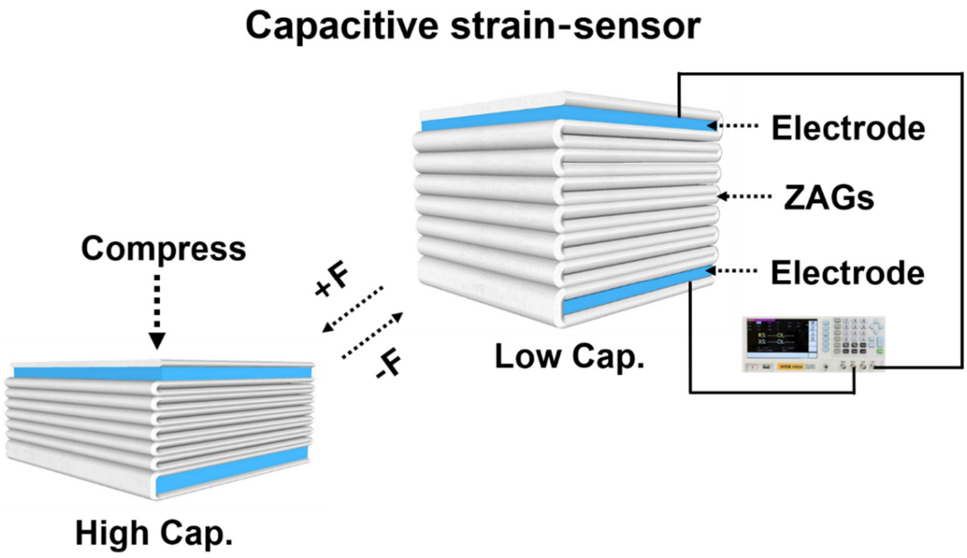

Supplementary Fig. 46 | Schematic illustration of the capacitive strain-sensor and its operation mechanism.

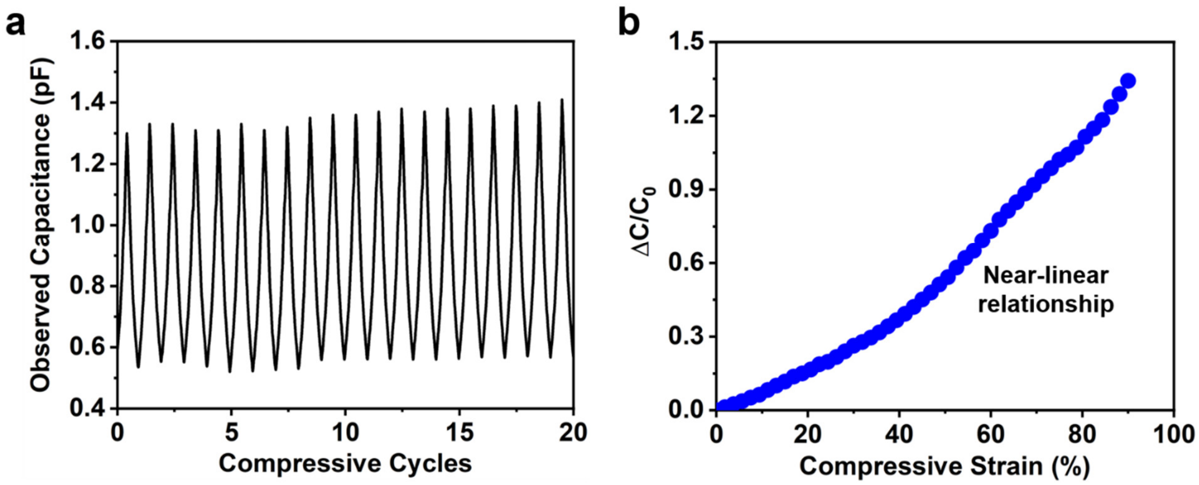

Supplementary Fig. 47 | Sensing property of ZAGs. a, The capacitance curve of the observed capacitance versus compressive cycles. **b**, The capacitance variation of strain-sensor with compressive strain.

Sample	Density (mg cm^{-3})	Calibration of κ_{air} (mW m^{-1} K^{-1})	$\kappa_{measured}$ (mW m^{-1} K^{-1})
1	15	25.99 --- 26.26	25.95
2	15		25.76
3	15		26.05
4	15		26.14
5	55		27.32
6	55		27.36
7	55		27.48
8	55		27.45

Supplementary Table 1 | Thermal conductivity of ZAGs with different densities.

Supplementary Video 1 | Compression test of ceramic aerogels. The compression properties were studied using samples with a height of 10 mm by an Instron 3365 at a loading rate of 2 mm min^{-1}.

Supplementary Video 2 | Fatigue test of ceramic aerogels. The fatigue properties were studied using samples with a height of 10 mm by an Instron 3365 at a loading rate of 2 mm min^{-1}.

Supplementary Video 3 | Tensile test of ceramic aerogels. The tensile properties were studied using samples with a length of 50 mm by an Instron 3365 at a loading rate of 2 mm min^{-1}.

Supplementary Video 4 | Bending test of ceramic aerogels. The bending properties were studied using samples with a length of 50 mm by an Instron 3365 at a loading rate of 2 mm min^{-1}.

Supplementary Video 5 | Thermal shock test of ceramic aerogels. The thermal shock tests were studied using a home-made pneumatic system in Supplementary Fig. 31 at a frequency of 0.07 Hz and temperature variation speed up to 200 °C s^{-1}.

Supplementary Video 6 | High-temperature thermal stability test of ceramic aerogels. The high-temperature thermal stability was tested using bilateral butane blowtorch flames (over 1,300 °C) under compression of 50% strain by an Instron 3365.

Supplementary Video 7 | Thermal insulation test of the aero engine (CFM56) fuel tube with ceramic aerogel barriers. The thermal insulating performances were tested on a typical CFM56 fuel tube with barrier thickness of 10 mm under a butane blowtorch flame (over 1,300 °C).

Supplementary Video 8 | Thermal insulation test under vibrating conditions of ceramic aerogels. The thermal insulating performance was tested with vibration amplitude to 30% of sample thickness at 0.25 Hz. The thermal profiles of the ZAGs were recorded by a Flir A615 when the sample was heated from the bottom at 400 °C under 100 times of vibration.

References

1. Itoh, T. Formation of polycrystalline zircon ($ZrSiO_4$) from amorphous silica and amorphous zirconia. *J. Cryst. Growth* **125**, 223-228 (1992).
2. Du, C., Yuan, Q. & Yang, Z. Lowering the synthesis temperature of zircon powder by yttria addition. *J. Mater. Sci. Lett.* **18**, 965-966 (1999).
3. Song, K. *et al.* Effect of ZrO_2 types on $ZrSiO_4$ formation. *Ceram. Int.* **45**, 23444-23450 (2019).
4. Bhattacharya, A. K., Hartridge, A. & Mallick, K. K. An X-ray diffraction and NMR study into the mechanism of zircon formation from aqueous sols. *J. Mater. Sci.* **31**, 5873-5876 (1996).
5. Jiang, Z. *et al.* Effect of Si/Zr molar ratio on the sintering and crystallization behavior of zircon ceramics. *J. Eur. Ceram. Soc.* **40**, 4605-4612 (2020).
6. Barducci, A., Bussi, G. & Parrinello, M. Well-tempered metadynamics: a smoothly converging and tunable free-energy method. *Phys. Rev. Lett.* **100**, 020603 (2008).
7. Steinhardt, P. J., Nelson, D. R. & Ronchetti, M. Bond-orientational order in liquids and glasses. *Phys. Rev. B* **28**, 784-805 (1983).
8. Ten Wolde, P. R., Ruiz-Montero, M. J. & Frenkel, D. Simulation of homogeneous crystal nucleation close to coexistence. *Faraday Discuss.* **104**, 93-110 (1996).
9. Ronneberger, I., Zhang, W., Eshet, H. & Mazzarello, R. Crystallization properties of the $Ge_2Sb_2Te_5$ phase-change compound from advanced simulations. *Adv. Funct. Mater.* **25**, 6407-6413 (2015).
10. Du, J., Devanathan, R., Corrales, L. R., Weber, W. J. & Cormack, A. N. Short- and medium-range structure of amorphous zircon from molecular dynamics simulations. *Phys. Rev. B* **74**, 214204 (2006).
11. Piaggi, P. M. & Parrinello, M. Entropy based fingerprint for local crystalline order. *J. Chem. Phys.* **147**, 114112 (2017).
12. Niu, H., Piaggi, P. M., Invernizzi, M. & Parrinello, M. Molecular dynamics simulations of liquid silica crystallization. *Proc. Natl. Acad. Sci. U.S.A.* **115**, 5348-5352 (2018).
13. Warren, B. E. X-ray Diffraction. (Courier Corporation, 1990).
14. Van Duin, A. C. T., Dasgupta, S., Lorant, F. & Goddard, W. A. ReaxFF: a reactive force field for hydrocarbons. *J. Phys. Chem. A* **105**, 9396-9409 (2001).
15. Van Duin, A. C. T. *et al.* $ReaxFF_{SiO}$ reactive force field for silicon and silicon oxide systems. *J. Phys. Chem. A* **107**, 3803-3811 (2003).
16. Hou, C., Xu, J., Ge, W. & Li, J. Molecular dynamics simulation overcoming the finite size effects of thermal conductivity of bulk silicon and silicon nanowires. *Modelling Simul. Mater. Sci. Eng.* **24**, 045005 (2016).

17. Wang, H. *et al.* High-temperature particulate matter filtration with resilient yttria-stabilized ZrO_2 nanofiber sponge. *Small* **14**, 1800258 (2018).
18. Jia, C. *et al.* Highly compressible and anisotropic lamellar ceramic sponges with superior thermal insulation and acoustic absorption performances. *Nat. Commun.* **11**, 3732 (2020).
19. Si, Y., Wang, X., Dou, L., Yu, J. & Ding, B. Ultralight and fire-resistant ceramic nanofibrous aerogels with temperature-invariant superelasticity. *Sci. Adv.* **4**, eaas8925 (2018).
20. Xu, X. *et al.* Double-negative-index ceramic aerogels for thermal superinsulation. *Science* **363**, 723-727 (2019).
21. Xu, X. *et al.* Elastic ceramic aerogels for thermal superinsulation under extreme conditions. *Mater. Today* **42**, 162-177 (2020).
22. Dou, L. *et al.* Temperature-invariant superelastic, fatigue resistant, and binary-network structured silica nanofibrous aerogels for thermal superinsulation. *J. Mater. Chem. A* **8**, 7775-7783 (2020).
23. Dou, L. *et al.* Interweaved cellular structured ceramic nanofibrous aerogels with superior bendability and compressibility. *Adv. Funct. Mater.* **30**, 2005928 (2020).
24. Zong, D. *et al.* Flexible ceramic nanofibrous sponges with hierarchically entangled graphene networks enable noise absorption. *Nat. Commun.* **12**, 6599 (2021).
25. Su, L. *et al.* Highly stretchable, crack-insensitive and compressible ceramic aerogel. *ACS Nano* **15**, 18354-18362 (2021).
26. Zhang, X. *et al.* Ultrastrong, superelastic, and lamellar multiarch structured ZrO_2-Al_2O_3 nanofibrous aerogels with high-temperature resistance over 1300 °C. *ACS Nano* **14**, 15616-15625 (2020).
27. Su, L. *et al.* Resilient Si_3N_4 nanobelt aerogel as fire-resistant and electromagnetic wave-transparent thermal insulator. *ACS Appl. Mater. Interfaces* **11**, 15795-15803 (2019).
28. Yin, J., Li, X., Zhou, J. & Guo, W. Ultralight three-dimensional boron nitride foam with ultralow permittivity and superelasticity. *Nano Lett.* **13**, 3232-3236 (2013).
29. Sun, Y. *et al.* A novel aerogels/porous Si_3N_4 ceramics composite with high strength and improved thermal insulation property. *Ceram. Int.* **44**, 5233-5237 (2018).
